# Cell type-specific interaction analysis using doublets in scRNA-seq

**DOI:** 10.1093/bioadv/vbad120

**Published:** 2023-09-06

**Authors:** Courtney Schiebout, Hannah Lust, Yina Huang, H Robert Frost

**Affiliations:** Department of Biomedical Data Science, Dartmouth College, Hanover, NH 03755, United States; MDI Biological Laboratory, Bar Harbor, ME 04609, United States; Department of Microbiology and Immunology, Dartmouth College, Hanover, NH 03755, United States; Department of Biomedical Data Science, Dartmouth College, Hanover, NH 03755, United States

## Abstract

**Summary:**

Doublets are usually considered an unwanted artifact of single-cell RNA-sequencing (scRNA-seq) and are only identified in datasets for the sake of removal. However, if cells have a juxtacrine interaction with one another *in situ* and maintain this association through an scRNA-seq processing pipeline that only partially dissociates the tissue, these doublets can provide meaningful biological information regarding the intercellular signals and processes occurring in the analyzed tissue. This is especially true for cases such as the immune compartment of the tumor microenvironment, where the frequency and the type of immune cell juxtacrine interactions can be a prognostic indicator. We developed Cell type-specific Interaction Analysis using Doublets in scRNA-seq (CIcADA) as a pipeline for identifying and analyzing biologically meaningful doublets in scRNA-seq data. CIcADA identifies putative doublets using multi-label cell type scores and characterizes interaction dynamics through a comparison against synthetic doublets of the same cell type composition. In performing CIcADA on several scRNA-seq tumor datasets, we found that the identified doublets were consistently upregulating expression of immune response genes.

**Availability and implementation:**

An R package implementing the CIcADA method is in development and will be released on CRAN, but for now it is available at https://github.com/schiebout/CAMML.

## 1 Introduction

Throughout the brief history of single-cell RNA-sequencing (scRNA-seq) analysis, instances where more than one cell has been recorded as a single cell (known as a doublet for two cells or a multiplet for two or more cells) are assumed to be technical artifacts and are only identified during quality control processing in order to be removed from downstream analyses (DePasquale *et al.* 2019, McGinnis *et al.* 2019, Bais and Kostka 2020). This is normally the correct course of action, as doublets can be an artifactual result of the cell dissociation protocol whereby two or more unrelated cells stick together (Bais and Kostka 2020); however, the indiscriminate removal of all cells that appear to be doublets begs the question: is biologically relevant information lost when these doublets are discarded? In other words, are some doublets actually the result of physically interacting cells that do not successfully separate during tissue dissociation steps? If so, these doublets could provide novel information regarding juxtacrine cell-to-cell interactions within an experimental model, which is normally challenging to study using scRNA-seq data.

Interaction analysis is an ongoing area of research in scRNA-seq analysis, particularly in the case of juxtacrine interactions where the cells interact with one another by physical contact, given that the loss of cell location information due to dissociation makes it very difficult to determine which individual cells may have been involved in interactions. Numerous methods have been put forth to address this challenge by adapting the results of interaction analysis to have lower resolution. Specifically, interaction analysis methods have been developed to give group- or cluster-level annotations, which identify interactions that are likely occurring between cells in whole clusters, such as SingleCellSignalR and CellChat (Cabello-Aguilar *et al.* 2020, Jin *et al.* 2021), or cell-level annotations, which identify when a certain cell has the expression profile befitting of sending or receiving a given interaction signal and can predict the other cell(s) involved but not identify them with complete certainty, such as NicheNet and ICELLNET (Browaeys *et al.* 2020, Noël *et al.* 2021). These methods are relatively robust, allowing investigators to perform differential interaction analysis between experimental models. However, given that neither type of method allows for the full identification of both cells involved in an interaction, they are inherently limited in their specificity. Furthermore, these methods do not take into account any of the doublets or multiplets potentially present in the data, but rather remove or ignore them in their analysis. Especially in the case of juxtacrine interactions, maintaining and analyzing physically interacting cells in the form of multiplets could be extremely informative for cell–cell interaction analysis (Burshtyn *et al.* 2000). Some researchers have recently explored the deliberate partial dissociation of tissues to enable the transcriptomic profiling of both singlets and interacting doublets (Boisset *et al.* 2018, Halpern *et al.* 2018). To date, these approaches have been limited to the identification of interacting cell types from the merged expression profile of the doublet. Although an improvement on earlier techniques, valuable information in the transcriptomic profile regarding the phenotypic impact of the interaction is ignored.

To address the challenges of interaction analysis using scRNA-seq data, an increasing number of interaction analysis methods have been developed that target spatial transcriptomics (ST) data, which maintains information on tissue architecture (Chen *et al.* 2015, Ståhl *et al.* 2016, Cang and Nie 2020, Dries *et al.* 2021). These allow cells in close proximity to be identified as such and analyzed with this context in mind. These methods are ideal for interaction analysis given their focus on maintaining cell location while performing transcriptomics, and serve as a useful gold standard for validating interactions inferred from methods like scRNA-seq that do not preserve location information; however, given that many datasets already exist in the scRNA-seq space that lack spatial information or validation, developing a method that can be used in these cases is a necessary and useful pursuit.

Here, we present Cell type-specific Interaction Analysis using Doublets in scRNA-seq (CIcADA), a method for discerning biologically relevant doublets and quantifying their interactions in scRNA-seq. We accomplish this by analyzing a joint scRNA-seq/Cellular Indexing of Transcriptomes and Epitopes by Sequencing (CITE-seq) dataset from the B16F10 mouse melanoma model that was intentionally partially dissociated in order to preserve potential cell–cell conjugates. This allowed us to investigate doublet identification in a dataset we were confident contained doublets and whose experimental model was of interest for immune cell interactions. We then further tested our results with publicly available scRNA-seq data to illustrate the robustness of our findings on datasets where doublets were not intentionally maintained. We further validated our approach with Visium (Chen *et al.* 2015) spatial data from the B16F10 model that recapitulates the findings from the scRNA-seq data. Throughout our investigation, we find that biologically relevant doublets can indeed be identified and that their resulting interactions are potentially clinically significant.

## 2 Materials and methods

### 2.1 CIcADA method

**Figure 1.**
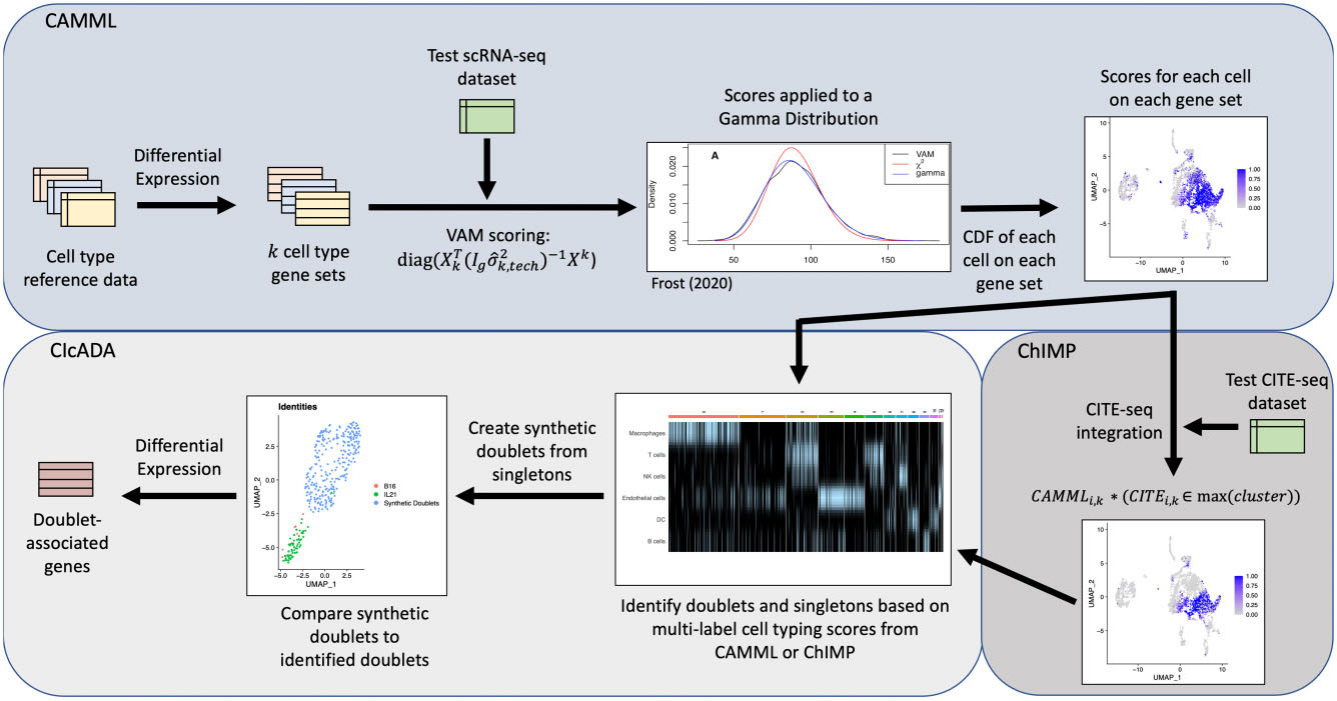
Flowchart of cell type and doublet identification steps.

The details of the CIcADA method are described in-depth in the following methods sections, but in brief, CIcADA carries out the following steps (and visualized in Fig. 1):

1. Perform cell type scoring on each of the cells in an scRNA-seq dataset using CAMML or ChIMP (depending on absence or presence of joint CITE-seq data) and identify doublets from cell types with notable overlap in the data.
2. Create synthetic doublets from high-confidence singletons to serve as a comparison for the detected doublets.
3. Cluster the synthetic and true doublets and contrast them using differential gene expression analysis.

This pipeline is explored using three datasets: a joint scRNA-seq/CITE-seq dataset from WT B16F10 and B16F10 IL-21 mouse melanoma with complementary Visium ST data, a joint scRNA-seq/CITE-seq dataset from a Mucosa-Associated Lymphatic Tissue (MALT) tumor available on 10X Genomics (10k Cells from a MALT Tumor 2018), and a scRNA-seq dataset comprised of lymphoma tumor cells also available on 10X Genomics (noa, b).

#### 2.1.1 Identify doublets with CAMML/ChIMP

To identify cells with a strong probability of being doublets, cell-type identification was performed using the ChIMP (CAMML with the Integration of Marker Proteins) method (Schiebout and Frost 2022b) on datasets containing joint scRNA-seq and CITE-seq data. For the datasets lacking CITE-seq data, cell-typing was performed using the CAMML (Cell-typing using variance Adjusted Mahalanobis distances with Multi-Labeling) method (Schiebout and Frost 2022a). CAMML performs multi-label cell typing of scRNA-seq data by using the variance-adjusted Mahalanobis (VAM) method to score each cell in the dataset for a collection of cell type-specific weighted gene sets (Frost 2020, Schiebout and Frost 2022a). The generated gene set scores, which are bound between 0 and 1, capture the association between each cell and the cell types of interest. ChIMP builds upon this process by adding an integration step for CITE-seq data whereby the CITE-seq counts for a given cell-type marker are separated by *k* = 2 *k*-means clustering (Schiebout and Frost 2022b). Cells with CITE-seq counts in the higher value cluster keep their CAMML score for the complementary cell type, while cells with CITE-seq counts in the lower value cluster are scored as zeroes (Schiebout and Frost 2022b). This CITE-seq integration step makes ChIMP more confident but also more conservative than CAMML. A workflow of CAMML, ChIMP, and CIcADA is visualized in Fig. 1.

In each dataset for this investigation, the gene sets used for cell-typing were built with celldex, whereby differential expression analysis of cell types of interest is performed with edgeR with a one versus all approach and genes with a log fold-change of greater than 5 are included in the relevant cell type gene set (Robinson *et al.* 2010, Aran *et al.* 2019). Cells are then scored with VAM, with genes weighted by their log2 fold-change value for that cell type. Cells that scored above a 0.75 for any two cell types were identified as potential doublets. This value was chosen given its inclusion of most high scores in the bimodal distribution of cell type scores across each dataset. Supplementary Fig. S3 illustrates the distributions of each cell type score in each dataset. Of note, this process inherently excludes the identification of homogeneous doublets in order to focus on interactions between differing cell types. Potential doublets were further filtered into groups based on the cell type composition, e.g. T cell/macrophage doublets, and the most notable doublet overlap was investigated. We did not attempt to identify and analyze higher-order multiplets given the small number of biological multiplets detected in the analyzed datasets, with fewer than 3% of cells in any dataset having more than two cell types with scores above 0.75.

#### 2.1.2 Separate artifactual and biological doublets

Once heterogeneous doublets have been identified, the next challenge is to determine if they are biological, i.e. cells undergoing a juxtacrine interaction, or just artifacts of the tissue and library preparation protocols used during scRNA-seq processing. Thus, a separate analysis step is needed to separate the heterogeneous doublets into biological and artifactual groups. To realize this separation, the CIcADA method compares the expression profile of identified heterogeneous doublets against synthetic doublets with the same cell type identities that are generated from singleton cells in the same dataset (the process for producing synthetic doublets is outlined in the following methods section). This procedure is carried out as follows: the raw counts from the identified heterogeneous doublets and synthetic doublets are merged into a single scRNA-seq dataset, processed, and clustered to separate doublets according to their cluster identification. These datasets are processed using the same steps as outlined in Section 2.2.3. For each unique cell type combination among the identified doublets, differential gene expression analysis is performed to identify transcriptomic differences between the biological and synthetic doublets.

#### 2.1.3 Run CAMML/ChIMP on singleton data and create synthetic doublets

To create synthetic doublets that represent a non-interacting benchmark for the separation of discern artifactual from biological doublets, we first identified high-confidence singletons in each scRNA-seq dataset using the cell type scores generated by ChIMP (Schiebout and Frost 2022b) or CAMML (Schiebout and Frost 2022a). Specifically, if a cell scored above 0.75 for one of the doublet cell types of interest and no higher than 0.5 for any other cell type, it was defined as a confident singleton. These values were selected for several reasons, above all because these cut-offs maintained power in the form of cell number without including notable amounts of noise and because they were robust to all datasets. These singleton cells were then combined with other singleton cells of the complementing cell type by summing their gene raw counts to produce synthetic doublets. For example, in the mouse B16F10 scRNA-seq/CITE-seq dataset, a population of macrophage and T cell doublets was identified by ChIMP (Schiebout and Frost 2022b). To create synthetic T cell/macrophage doublets, singleton T cells and macrophages were identified as cells in the B16F10 data that scored above 0.75 via the ChIMP method for either macrophage or T cell and no greater than 0.5 for any other cell type. These singletons were randomly combined by summing their raw gene counts to create synthetic T cell/macrophage doublets. Because both synthetic and artifactual doublets capture a combined transcriptome in the absence of juxtacrine interactions, we expect them to have similar mean expression profiles. While creating synthetic doublets in order to identify doublets in a dataset is not a new concept, prior methods do so without regard for the cell types present, inhibiting any investigation of their biological significance as it relates to specific cell–cell interactions (McGinnis *et al.* 2019, Bais and Kostka 2020).

#### 2.1.4 Separate experimental and synthetic doublets by clustering

The raw gene expression counts for the doublets identified in the experimental data and the synthetic doublets are built into Seurat datasets and merged (Satija *et al.* 2015). Each object is filtered to retain cells with at least 100 genes and genes present in at least 50% of cells in that dataset. Following the merge of the datasets, mitochondrial and ribosomal genes are removed for clarity in downstream differential expression analysis. Following this, the count data are transformed by natural-log transformation and centered and scaled (Satija *et al.* 2015). The data are finally clustered and visualized on 30 principal components with the same parameters as the original datasets, i.e. shared nearest neighbors clustering at a resolution of 0.25 and visualization of the first two Uniform Manifold Approximation Projection (UMAP) dimensions (Satija *et al.* 2015, McInnes *et al.* 2018). Of note, a low clustering resolution was used in this analysis to be consistent with the hypothesis that two groups would be present; however, other clustering resolutions were tested in order to confirm that no spurious clustering was occurring (Supplementary Fig. S4). In every case, higher cluster resolution did not result in clusters with mixed doublet populations. Rather, the existing cluster architectures split into smaller groups, which do not affect downstream analysis steps. Following this, the clusters are evaluated relative to original identities of the doublets, either found in the experimental data or synthetic, and only doublets in clusters entirely made of experimental doublets are considered to be biological doublets and kept for further analysis. This is an intentionally stringent approach for filtering in order to maintain the highest likelihood that the analyzed doublets represent interacting cells. The biological doublet clusters are then compared with synthetic doublet clusters using differential gene expression analysis to discern the meaningful biological impact of the potential juxtacrine interaction. Additionally, differential gene expression analysis was also performed between hypothesized doublets that clustered with synthetic doublets and their synthetic doublet counterparts in order to ensure there were no clustering misidentifications. The top genes for this analysis are available in Supplementary Table S4.

### 2.2 B16F10 data processing

#### 2.2.1 B16F10 and B16F10 IL-21 tumor induction and tumor infiltrating leukocytes isolation

Six- to 8-week-old C57BL/6 were injected subcutaneously with 5 × 105 B16F10 parental or B16F10 cells constitutively expressing IL-21 (B16F10 IL-21) on the right flank. Tumors were excised, smashed, and filtered through a 70-μm cell strainer to create a cell suspension prior to separation on a 40%–80% percoll (GE) gradient. Viable tumor infiltrating leukocyte (TIL) cell layers were collected for scRNA-seq and CITE-seq analysis. Single-cell RNA-seq and CITE-seq processing and transcript quantification were carried out using Cell Ranger from 10X Genomics (Zheng *et al.* 2017). The following antibodies were used for CITE-seq: CD4, CD8b, CD3, CD45, NK1.1, Thy1.2, CD25, CD11b, CD19, CD11c, CD204, and IL-23R (antibody panel details are available in Supplementary Table S1).

#### 2.2.2 Visium 10X Genomics ST data processing

Two B16F10 and two B16F10-IL-21 tumors were excised and separated from skin prior to embedding in optimal cutting temperature, snap freezing cryomolds in liquid nitrogen, and storage at −80°C. Tumors were cryo-sectioned by the Dartmouth Health Pathology Core facility and transferred to a Visium Spatial slide. The Visium Spatial slides were then aligned, processed, and quantified with Space Ranger from 10× Genomics (noa, a).

#### 2.2.3 scRNA-seq/CITE-seq data processing

**Figure 2.**
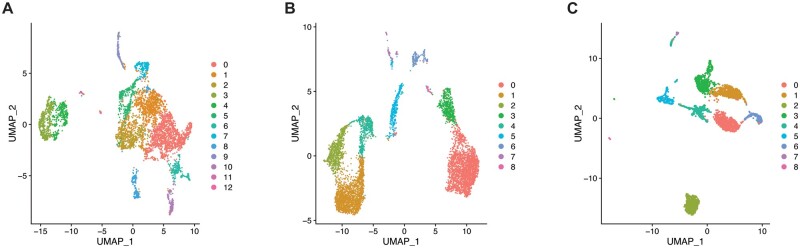
Visualization of the UMAPs and clusters for each of the analyzed datasets. UMAPs colored by cluster of (A) the B16F10 melanoma dataset, (B) the MALT dataset, and (C) the lymphoma dataset.

The joint scRNA-seq/CITE-seq data collected from the B16F10 mouse model of melanoma were first merged to include data from both the WT and IL-21+ tumors. This merged data were then filtered to only include genes present in at least 10 cells and to only include cells with at least 100 genes. Cells with more than 5% mitochondrial genes were also excluded to avoid capturing damaged or dying cells. The scRNA-seq data were natural-log transformed and scaled (Satija *et al.* 2015). The CITE-seq data were normalized by centered log ratio transformation and centered and scaled in the same manner as the transcriptomic data (Satija *et al.* 2015). The scRNA-seq was then clustered by shared nearest neighbors with “FindNeighbors” and “FindClusters” in Seurat with a resolution of 0.25 and visualized with UMAP on 30 principal components (Satija *et al.* 2015, McInnes *et al.* 2018). The parameter of 30 principal components was selected in part to stay consistent with common user defaults, as 30 is often utilized in scRNA-seq pipelines, and to be relatively conservative. This resulted in 5223 cells in 13 clusters with data for 16 248 genes (Fig. 2A).

#### 2.2.4 ST data processing

Four slides of tumors were collected and analyzed using the 10× Visium ST platform (Ståhl *et al.* 2016) for the same B16F10 model used to collect the scRNA-seq/CITE-seq data. Two slides were tumors of the wild-type (WT) B16F10 melanoma model and two were tumors of the B16F10 melanoma model with constitutive expression of IL-21. The resulting ST data were processed and filtered using Seurat (Satija *et al.* 2015). Each slides’ expression data were scaled and transformed using SCTransform on 3000 variable features (Hafemeister and Satija 2019). The data were then clustered with “FindNeighbors” and “FindClusters” in Seurat using a resolution of 0.25 on 30 principal components and visualized via UMAP (Satija *et al.* 2015, McInnes *et al.* 2018). After processing, the first WT slide had 12 967 genes across 3107 spots and the second WT slide had 12 181 genes across 2797 spots. The IL-21 slides had 12 670 genes across 2189 spots and 10 292 across 1263 spots, respectively.

#### 2.2.5 Confirmation of B16F10 T cell/macrophage interactions using ST data

The spots for each B16F10 Visium slide were analyzed with multi-label CAMML to identify co-localization of cell types (Schiebout and Frost 2022a). The gene sets used to estimate the cell type profile for each spot were built based on differential gene expression analysis from the ImmGen dataset in celldex on six cell types: macrophages, T cells, NK cells, B cells, dendritic cells (DCs), and endothelial cells, the same cell types used in the scRNA-seq analysis for the B16F10 model (Heng *et al.* 2008, Aran *et al.* 2019). Genes that had a log2 fold-change of greater than 3 for a given cell type were included in the gene set for that cell type and weighted by the log2 fold-change value. The log2 fold-change cut-off was lowered for the Visium data relative to scRNA-seq data due to increased sparsity. CAMML was then run on each of the Visium slides to determine which spots represented a mixture of different cell types (Schiebout and Frost 2022a). The CAMML generated cell type scores were then used to determine the rate of co-localization of potential doublet pairs and confirm doublet patterns identified in the scRNA-seq/CITE-seq data. Co-localization was defined as spots where each of the cell types of interest accounted for at least 10% of the sum of all cell-type CAMML scores for that spot (T cells or macrophages in this case). To determine if this rate of co-localization was greater than what would be expected for a slide, random permutations were carried out whereby the CAMML scores for each cell type in each spot were randomized without replacement and co-localization was recalculated on the randomized data. In other words, every CAMML score for T cells, for example, was randomly assigned to a single spatial spot, then the same was done for every other cell type. The true rate of co-localization was then compared with the distribution of 1000 co-localization rates when CAMML scores were permuted. Moran’s *I* was also computed for each slide using a binary of the co-localization rate versus spot location (Moran 1950).

### 2.3 Additional dataset processing

The same steps for scRNA-seq and CITE-seq processing performed on the B16F10 data were carried out for two additional freely available public scRNA-seq datasets from a MALT tumor (10k Cells from a MALT Tumor 2018) and a human lymphoma sample (noa, b), with some minor exceptions. Both datasets were sensitive to filtering by mitochondrial genes, and thus, to maintain cell counts, the cut-off for exclusion was set to cells with more than 10% of genes being mitochondrial. Additionally, the human lymphoma dataset did not include CITE-seq data, so the steps specific to processing that omics modality were skipped. Following these processing steps, the MALT data had 6438 cells over nine clusters with data for 15 518 genes (Fig. 2B) and the human lymphoma data had 2717 cells across nine clusters with gene expression counts for 16 445 genes (Fig. 2C).

## 3 Results and discussion

### 3.1 Doublet identification

**Figure 3.**
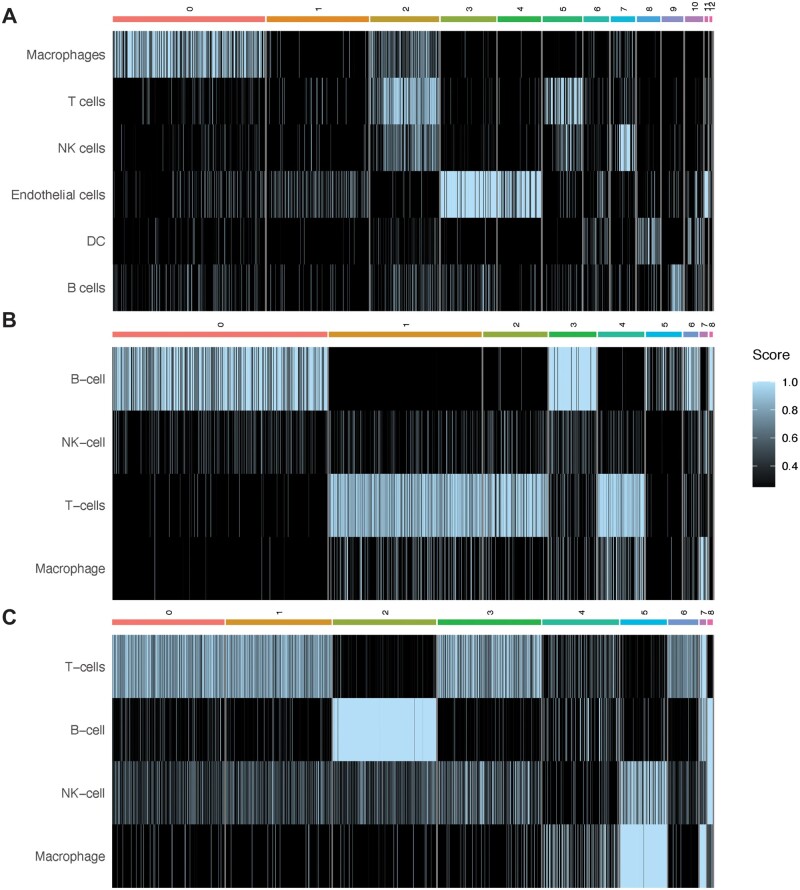
Heatmaps of the ChIMP/CAMML scores for each scRNA-seq dataset. (A) The ChIMP cell type scores for the B16F10 data, with notable overlap of T cells and macrophages in cluster 2. (B) The ChIMP cell type scores for the MALT data, with notable overlap of T cells and macrophages in cluster 4. (C) The CAMML cell type scores for the lymphoma data, with NK cell and macrophage overlap in cluster 5. Columns correspond to the clusters identified in each dataset, which can be visualized in Fig. 2.

ChIMP (or CAMML for the dataset of lymph node cells that did not have CITE-seq data) was used to perform multi-label cell typing of the analyzed single cell transcriptomics data for common immune cell types (Schiebout and Frost 2022a,b). In the B16F10 model of mouse melanoma, ChIMP analysis was performed for six cell types: macrophages (CD11b+), T cells (CD4+ or CD8+), NK cells (NK1.1+), endothelial cells (CD45−), DCs (CD11c+), and B cells (CD19+) (Zhang *et al.* 2019, Schiebout and Frost 2022b). Following ChIMP analysis, cluster 2 was identified as having the highest overlap of scores greater than 0.75, with macrophages and T cells having the highest average scores in this cluster (0.36 and 0.67, respectively), although NK cells also scored highly, with an average of 0.35 (Fig. 3A). T cells and macrophages were therefore used to evaluate potential juxtacrine interactions that may have resulted in doublet formation. It was found that 69 cells met the cut-offs for cell-type co-expression required for classification as T cell/macrophage doublets. Of note, 61 of these 69 doublets originated from the IL-21+ data.

In the MALT dataset, ChIMP was executed for four cell types: B cells (CD19+), NK cells (CD56+), T cells (CD4+ or CD8+), and macrophages (CD14+) (10k Cells from a MALT Tumor 2018, Zhang *et al.* 2019, Schiebout and Frost 2022b). Some cell types used in the B16F10 ChIMP analysis were excluded from the analysis for this dataset due to the differing markers used for CITE-seq. In this dataset, there was also a predominant overlap in cell type scores between T cells and macrophages, particularly in cluster 4 (Fig. 3B). When these cells were analyzed for scoring overlap, 385 cells were identified as potential T cell/macrophage doublets.

Finally, in the lymphoma dataset, the same four standard cell types used in the MALT ChIMP analysis were used for CAMML scoring: B cells, NK cells, T cells, and macrophages (noa, b; Schiebout and Frost 2022a). As mentioned previously, this dataset does not have a paired CITE-seq dataset, so only scRNA-seq was used for cell typing. In this case, the greatest overlap of cell types scoring above 0.75 was in cluster 5, where both NK cells and macrophages were highly represented (Fig. 3C), with 155 cells identified as potential NK cell/macrophage doublets.

Given our use of public data, the live cell rate cannot be accessed to further validate the number of doublets identified; however, the cell pairs identified as potential doublets are consistent with prior research on the tumor microenvironment. In particular, macrophages can be instrumental in activating or inhibiting both T cells and NK cells by releasing cytokines and can also be acted upon, in the form of activation or inhibition, by both T cells and NK cells (Bellora *et al.* 2010, Ansell and Vonderheide 2013, Zhou *et al.* 2021).

### 3.2 Doublet separation

**Figure 4.**
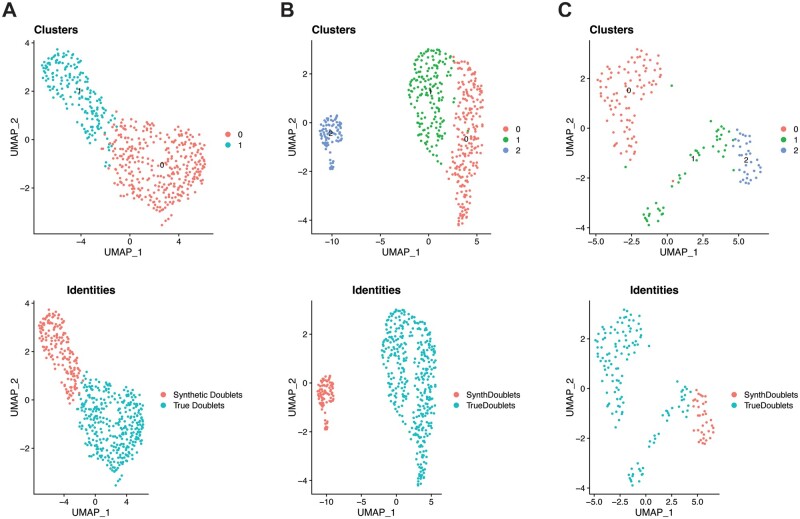
UMAP projections for each of the analyzed datasets. UMAPs of the (A) B16F10, (B) MALT, and (C) lymphoma datasets showing the clustering of true and synthetic doublets in the top row and their actual identities in the bottom row.

Following identification of doublets using cell-typing, synthetic doublets were created for each dataset for comparison. In the B16F10 dataset, 279 high-confidence singleton T cells and 685 high-confidence singleton macrophages were identified. The number of unique synthetic doublets that can be created is limited by the size of the smaller cell type population, in this case 279 T cells. The raw count data for the synthetic doublets were then merged with the count data for the identified biological doublets and the merged dataset was normalized, clustered, and visualized via UMAP using the same processing steps and with the same parameters as outlined in Section 2.2.3 (Satija *et al.* 2015, McInnes *et al.* 2018). UMAP visualizations of both the clustering of these doublets and their true identities are shown in Fig. 4A.

The same synthetic doublet creation process was carried out for the MALT and lymphoma datasets (10k Cells from a MALT Tumor 2018; noa, b). The MALT data had 1459 high-confidence T cell singletons and 88 high-confidence macrophage singletons, resulting in 88 synthetic doublets. The clustering and original identities of these synthetic doublets and the doublets identified by cell-typing with ChIMP are visualized in Fig. 4B. The lymphoma dataset had 39 high-confidence NK cell singletons and 158 high-confidence macrophage singletons, giving 39 synthetic doublets. These synthetic doublets clustered and visualized against the doublets identified with cell-typing by CAMML are visualized in Fig. 4C.

As seen in Fig. 4, most of the synthetic doublets and doublets identified by cell-typing with ChIMP or CAMML separate by clustering. This is not surprising given the stringent set of cut-offs used to identify both cell-typed doublets and high-confidence singletons and to include genes in the respective datasets. However, there are some cell type-specific doublets that do cluster with synthetic doublets, indicating that artificial doublets can be effectively identified should they pass through the aforementioned exclusionary steps. This highly stringent process for identifying doublets then allows us to evaluate how these doublet groups differ from one another and the biological implications of these distinct transcriptomic profiles.

Given that the volume of completely unique synthetic doublets that can be created is limited by the number of high-confidence singletons, this same analysis was repeated in each dataset with 1000 synthetic doublets created by randomly selecting a singleton of each cell type 1000 times and combining them. This analysis resulted in similar differential gene expression results to the original. The clustering for this analysis is available in Supplementary Fig. S2 and the top differentially expressed genes are available in Supplementary Table S3.

### 3.3 Doublet differential expression

Following the separation of doublets by clustering, resulting in the exclusion of any doublets that clustered with synthetic doublets, differential gene expression analysis was performed with “FindAllMarkers” in Seurat to compare the cluster of doublets identified by cell-typing with ChIMP to all other clusters present in the data (Satija *et al.* 2015). In the B16F10 dataset, several genes with implications in immune response to cancer were upregulated in this group, including Cxcl9, interferon gamma, and Gpb2b (Arlauckas *et al.* 2018, Pascual-García *et al.* 2019, Jorgovanovic *et al.* 2020). True doublets also express significantly higher amounts of H2-K1 and H2-D1, both of which are MHC genes associated with juxtacrine interaction in the form of antigen presentation to T cells (Ohtsuka *et al.* 2008). A table of the most upregulated genes in each group is available in the Supplementary Information (Supplementary Table S2).

The differential expression analysis yielded similar results in the two validation datasets as well. The MALT dataset’s doublets had upregulation of immune response genes, including TXNIP and ETS1 (Chen *et al.* 2020, Kim *et al.* 2020). There were also several notable genes associated with the T-cell receptor upregulated in the true doublets relative to their synthetic counterparts, including TRAC, TRBC2, and several HLA genes, although the adjusted *P*-value of these genes varied in significance. The lymphoma dataset had genes related to NK cell and macrophage immunity, such as CXCL8 and MRC1, although interestingly, in this case, the roles of the upregulated genes represented both pro- and anti-tumoral immune responses (Montaldo *et al.* 2012, Arlt *et al.* 2020, Walle *et al.* 2022). Furthermore, CD48, a gene commonly associated with juxtacrine interaction between NK cells and myeloid cells, has about double the expression in true doublets versus synthetic doublets, although this association is not significant when the *P*-value is adjusted (Mattiola *et al.* 2015). A table with all of the top upregulated genes in each of these datasets is available in the Supplementary Information (Supplementary Table S2).

Collectively, these results indicate that utilizing multi-label cell-typing as a means of identifying potential biological doublets is effective and can provide important information regarding immune cell interactions with the tumor microenvironment. In all three cases, the genes found to be upregulated in the doublets identified using cell-typing, relative to synthetic doublets of the same cell types, are biologically relevant and informative for understanding how immune cells may be collaborating in response to cancer.

### 3.4 ST validation

**Figure 5.**
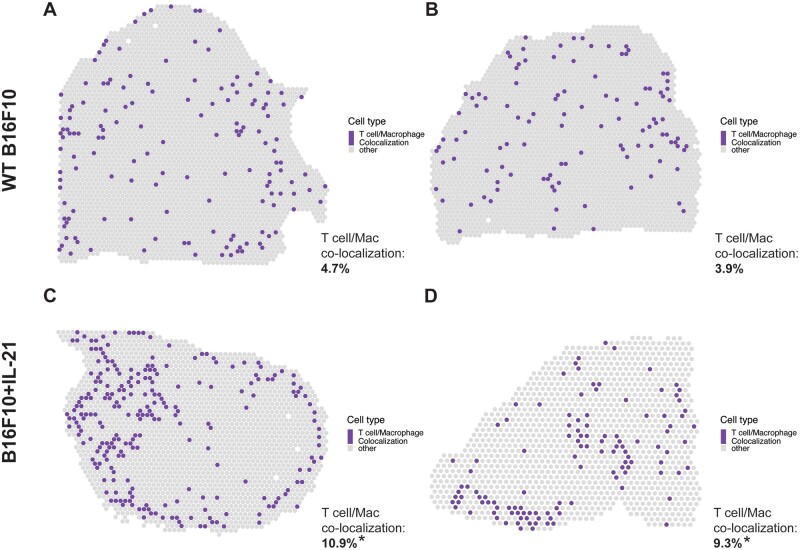
Spatial plots showing the proportions of T cell and macrophage CAMML scores at each Visium spot in the B16F10 data. Co-localization is the percent of spots where T cell and macrophage cell type scores each make up at least 10% of the score for that spot. Significance is determined based on the empirical CDF of random permutation of cell type scores across spots for that slide. Moran’s *I* observed values are also reported for each slide based on the spatial autocorrelation of the co-localization metric. The top two panels (A and B) are WT B16F10 tumors and the bottom two panels (C and D) are tumors from B16F10 with constitutively active IL-21. The percent of spots where both T cells and macrophages scores make up at least 10% of all CAMML scores for that spot is provided in the bottom right of each panel. Spatial plots were built using the “plotSpatialScatterpie” function from SPOTlight (Elosua-Bayes *et al.* 2021).

**Figure 6.**
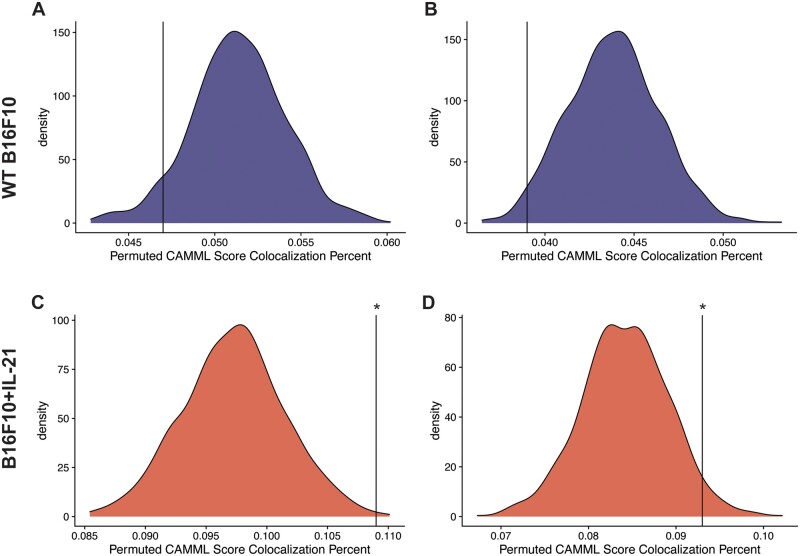
Density plots for the permuted distributions of co-localization statistics for each of the ST slides. Density plots (A) and (B) are WT B16F10 slides 1 and 2, respectively, and density plots (C) and (D) are the B16F10 + IL-21 slides 3 and 4. In each case, the black vertical line represents the true co-localization percent for that slide, with significance determined based on the empirical CDF of the permuted values.

To further validate our findings, we utilized ST data generated using the 10× Visium technology on the same B16F10 mouse model of melanoma analyzed via joint scRNA-seq/CITE-seq (Ståhl *et al.* 2016). Specifically, the ST data were used to determine if the findings in the joint scRNA-seq/CITE-seq data could be detected in a different modality. We found a notable increase in the co-localization (as outlined in the “Confirmation with spatial transcriptomic data” methods section) of T cells and macrophages in the ST data generated on IL-21+ tumors, which supports the scRNA-seq findings where only 8 and the 69 biological doublets originated from the WT data (Fig. 4). We further tested this finding with Moran’s *I* on the co-localization status of each spot (Moran 1950). Every slide was significant for spatial autocorrelation with Moran’s *I* but the observed value for the IL21+ tumors was about an order of magnitude higher than the observed value in the WT tumors (Fig. 5). Furthermore, following permutation of the CAMML scores, which serves to provide a permutation null distribution of the expected co-localization rates (see the “Confirmation with spatial transcriptomic data” section of the methods for details), we found that the rate of co-localization in the IL-21+ ST data was significantly higher than what would be expected by chance (according to a *P* < .05 on the empirical CDF of the permuted distributions). Interestingly, the WT tumors presented the opposite trend, where the rate of co-localization was actually lower than the average of the permuted distribution (Fig. 6). Overall, these findings support the doublet analysis, with notable co-localization of T cells and macrophages, particularly in the cells originating from IL-21+ data.

**Figure 7.**
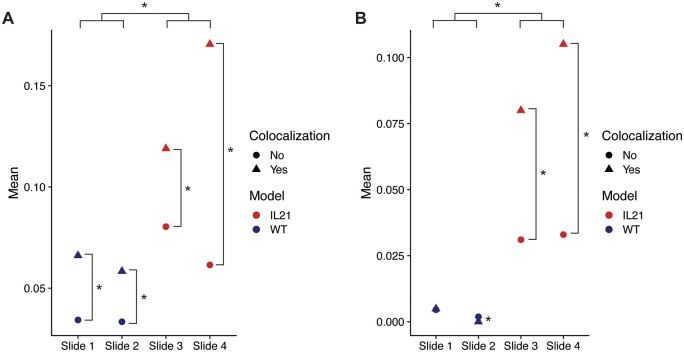
Average expression of the Cxcl9 and Gpb2b genes per spot in Visium slides from the B16F10 model of mouse melanoma. Points are split by co-localization status (true when macrophage and T cell scores account for at least 10% of all CAMML scores for that spot) and slide number. Slides 1 and 2 represent WT B16F10 tumors and slides 3 and 4 represent B16F10 tumors with constitutively active IL-21, as designated by color. (A) Average gene expression per spot of Cxcl9. (B) Average gene expression per spot of Gbp2b. Significance between points was determined using the Wilcoxon Rank Sum Test (Wilcoxon 1945).

To further validate the scRNA-seq findings, the upregulated genes found using differential expression analysis were evaluated using the ST data. Although some of the top genes could not be evaluated given the sparsity of ST data, Cxcl9 and Gbp2b both had non-zero ST expression data. In both cases, the average expression was significantly increased in spots that had co-localization of T cells and macrophages relative to spots that did not (Fig. 7). Furthermore, the average expression of both genes was significantly increased in the IL-21+ tumor slides relative to the WT slides (Fig. 7). These results further support the associations deduced from the scRNA-seq doublet analysis. Importantly, this validation is based on an independent transcriptomics modality, 10× Visium ST, that is particularly useful for evaluating cell–cell interactions since it maintains tissue architecture. Additionally, these findings recapitulate existing knowledge about the B16F10+IL-21 model of mouse melanoma, specifically that it has higher immune activation and smaller tumor size relative to the WT model.

## 4 Conclusion

Doublets occurring in scRNA-seq data have historically been treated as artifactual errors to be identified and removed before downstream analyses (DePasquale *et al.* 2019, McGinnis *et al.* 2019, Bais and Kostka 2020). However, this approach overlooks the reason for doublet persistence in typical scRNA-seq dissociation protocols and their potential biological relevance. To evaluate scRNA-seq doublets, and to discover if they hold meaningful information regarding juxtacrine interactions, we developed the CIcADA method. CIcADA is a bioinformatics pipeline for identifying heterogeneous doublets in scRNA-seq data, separating doublets into artifactual and biological populations, and investigating the biological doublets for information regarding juxtacrine signaling within the source tissue.

We evaluated this method on a locally generated B16F10 scRNA-seq/CITE-seq dataset where only partial dissociation was carried out to improve the likelihood of maintaining interacting cell pairs. In this dataset, CIcADA successfully identifies single cell data points that have high doublet potential and, when compared with synthetic doublets, these candidate doublets showed high expression of genes associated with immune cell interaction in the tumor microenvironment. The findings from the scRNA-seq/CITE-seq analysis were validated using 10× Visium ST data measured on the same murine B16F10 tumor model and the co-localization of cell types and gene expression analyses confirmed the signals detected in the scRNA-seq data (Ståhl *et al.* 2016). Additional analysis of public scRNA-seq datasets that underwent the typical tissue dissociation process illustrates that our method is still effective at finding doublets with biologically relevant implications in cases where doublet maintenance was not a priority. CIcADA is a stringent method for detecting and evaluating biologically relevant doublets in scRNA-seq data, with a particular emphasis for utilization on scRNA-seq data of the tumor microenvironment.

## Supplementary Material

vbad120_Supplementary_Data

## Data Availability

The B16F10 data is pending submission to GEO. An update with the necessary accession number will be provided when it is available. For now, anyone seeking to utilize the data may contact the corresponding author via email. The MALT dataset is available from 10X Genomics at https://www.10xgenomics.com/resources/datasets/10-k-cells-from-a-malt-tumor-gene-expression-and-cellsurface-protein-3-standard-3-0-0. The lymphoma dataset is also available from 10X Genomics at https://www.10xgenomics.com/resources/datasets/hodgkinslymphoma-dissociated-tumor-whole-transcriptome-analysis-3-1-standard-4-0-0.
