# Supplementary material for: Cell type-specific interaction analysis using doublets in scRNA-seq
Source: Bioinform Adv. 2023 Sep 6;3(1):vbad120. doi: 10.1093/bioadv/vbad120 (PMC10516525; doi:10.1093/bioadv/vbad120)
Supplement: vbad120_Supplementary_Data [file vbad120_supplementary_data.pdf]

Supplementary Information:

1. Negative Control Methods

In order to account for cases when there are no true doublets in a dataset, a negative control case was tested. In this case, two different sorted cell types (monocytes and naive CD4 T cells) from Zheng, et al. (2017) (1) were combined and CIcADA was performed. This data was processed in the same way as the prior datasets, the process of which is outlined in the "scRNA-seq/CITE-seq data processing" section of the Methods. After this process, 13,037 cells with 12,076 genes remained for analysis. CAMML was performed with gene sets developed by the same process as the prior datasets: with differential gene expression of the Human Primary Cell Atlas data for monocytes and T cells. Genes with a log2 fold-change of 5 or greater were kept and their log2 fold-change was used for weighting in CAMML. Following CAMML scoring, 97 cells scored above a 0.75 for both cell types. Relative to the volume of cells, this was an extremely small overlap. Additionally, neither cell type had perfect sorting, with 98% purity in both cell types, so slight cell overlap is not completely unexpected (1). 3,969 cells scored as highly confident T cell singlets, and 2,602 cells scored as highly confident monocyte singlets, so 2,602 synthetic doublets were created by random combination of singletons for comparison to the 180 suspected doublets. The clustering of these populations, performed in the same way as outlined for the three test datasets, is shown in Supplementary Figure 1. Of note, the suspected doublets do slightly separate from the synthetic doublets, but the physical association is close. Given its small relative size and its minimal separation in UMAP, this investigation validates that CIcADA will not falsely identify doublet populations in datasets that lack them.

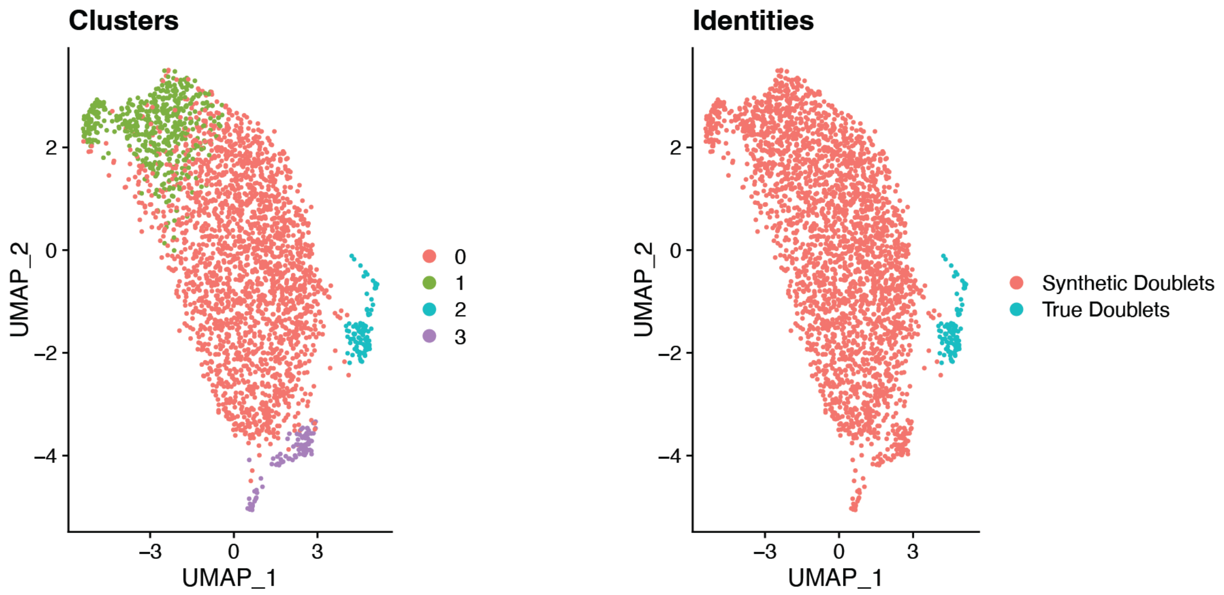

Supplementary Figure 1. UMAP projections for the negative control dataset's suspected doublets compared to synthetic doublets. The UMAP on the left shows clustering of identified doublets and synthetic doublets, and the UMAP on the right shows their actual identities.

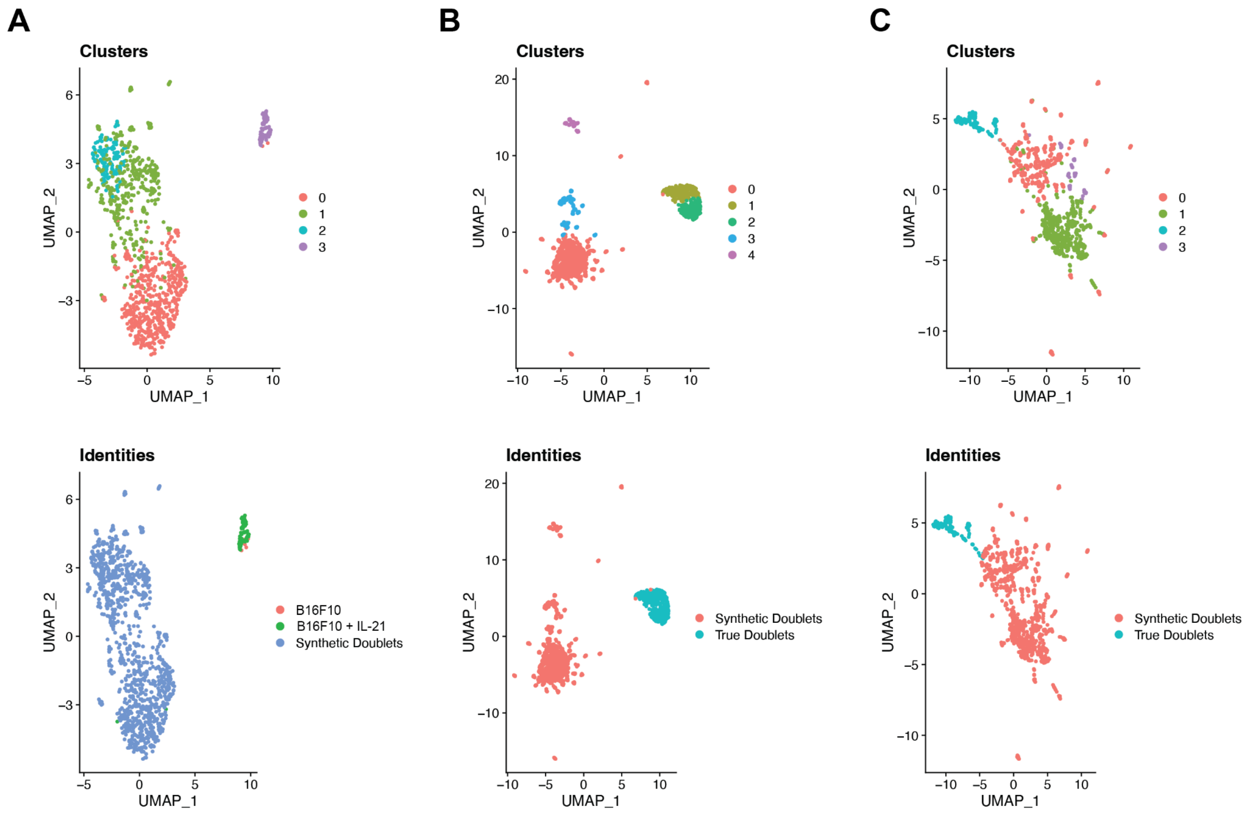

Supplementary Figure 2. UMAP projections for each of the analyzed datasets compared to 1,000 synthetic doublets. UMAPS of **A.** B16F10, **B.** MALT, and **C.** lymphoma showing the

clustering of true doublets and 1,000 synthetic doublets in the top row and their actual identities in the bottom row.

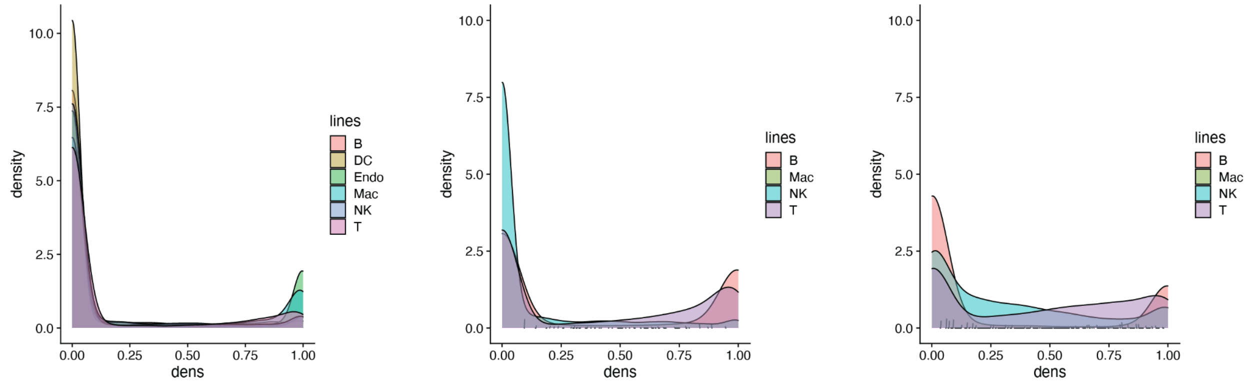

Supplementary Figure 3. Density plots of the distribution of cell-type scores for each cell in the **A.** B16F10, **B.** MALT, and **C.** lymphoma datasets. An upper limit of 10 was set on the y-axis for **B.** and **C.** given the stark binary distribution of macrophage scores, which suppresses visualization of the other cell type distributions.

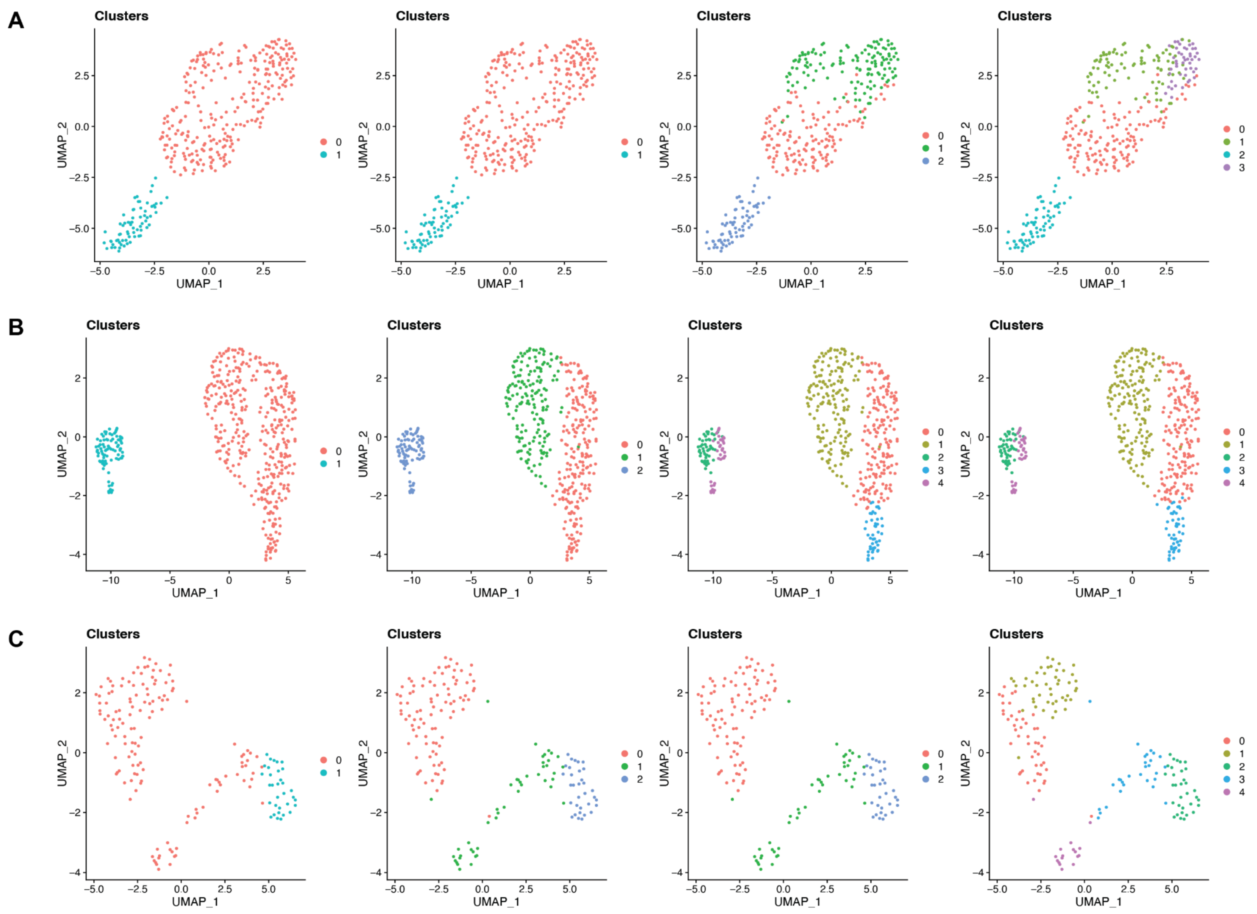

Supplementary Figure 4. UMAP plots of the clustering of true and synthetic doublets in the **A.** B16F10, **B.** MALT, and **C.** lymphoma datasets. From left to right, the following cluster resolutions were tested: 0.05, 0.25, 0.5, and 0.75.

Marker	AB	Catalog #
CD4	TotalSeq™-A0001 anti-mouse CD4 Antibody	100569
CD8b	TotalSeq™-A0230 anti-mouse CD8b (Ly-3) Antibody	126623
CD3	TotalSeq™-A0182 anti-mouse CD3 Antibody	100251
CD45	TotalSeq™-A0096 anti-mouse CD45 Antibody	103159
NK1.1	TotalSeq™-A0118 anti-mouse NK-1.1 Antibody	108755
Thy1.2	TotalSeq™-A0075 anti-mouse CD90.2 Antibody	105345
CD25	TotalSeq™-A0097 anti-mouse CD25 Antibody	102055
CD11b	TotalSeq™-A0014 anti-mouse/human CD11b Antibody	101265
CD19	TotalSeq™- A0093 anti-mouse CD19 Antibody	115559
CD11c	TotalSeq™-A0106 anti-mouse CD11c	117355
CD204	TotalSeq™-A0448 anti-mouse CD204	154703
IL-23R	TotalSeq™- A1033 anti-mouse IL-23R Antibody	150909

Supplementary Table 1. B16F10 mouse model of melanoma CITE-seq antibody panel.

Dataset	Upregulated doublet genes
B16F10 (WT/IL-21)	Hmox1, Ccl5, Fcgrt, Klre1, Serpinb6b, Gpb2b, Cxcl9, Il18rap, Nr1h3, Ifng
MALT	ARID5B, RNF19A, TXNIP, TRAC, CD3G, TRBC2, ITM2A, CD2, ETS1, CD3D
Lymphoma	CXCL8, MRC1, LIPA, IL1RN, CLEC10A, EREG, C5AR1, G0S2, IL1B, F13A1

Supplementary Table 2. Top 10 upregulated genes in the identified doublet clusters compared to the synthetic doublet clusters for each dataset.

Dataset	Upregulated doublet genes
B16F10 (WT/IL-21)	Hmox1, Ckb, C1qc, Fcgrt, Klre1, Serpinb6b, Gpb2b, Ifi44, Nr1h3, Ifng

MALT	IL32, CD3G, DUSP4, TRAC, NR3C1, ARID5B, CD2, RNF19A, CD3D, ITM2A
Lymphoma	S100A9, IL1B, CXCL2, CXCL8, THBS1, EREG, MS4A6A, C5AR1, G0S2, CEBPD

Supplementary Table 3. Top 10 upregulated genes in the identified doublet clusters compared to the synthetic doublet clusters for each dataset when 1,000 doublets are created.

Dataset	Upregulated doublet genes
B16F10 (WT/IL-21)	Napsa, Dusp6, Gsr, Tnfsf13, Mbp, Atf7, Ms4a8a, Ano6, Cfl2, Hsd3b7
Lymphoma	GADD45B, HSP90B1, HSPD1, MCL1, JUNB, NFKBIA, SRP14, CHCHD2, ZFP36, TAGLN2

Supplementary Table 4. Top 10 upregulated genes in hypothesized doublets that cluster with synthetic doublets relative to their synthetic counterparts.

Cell Type	Genes	CITE-seq
B cells	Ms4a1 (9.46), Cr2 (8.74), Cd19 (8.28), Pax5 (8.09), Fcer2a (8.05), Fcmr (7.85), Cd79a (7.64), Ebf1 (7.25), Ighd (7.2), Pou2af1 (6.72), Blk (6.71), Chst3 (6.5), Cd79b (6.5), Scd1 (6.45), Cecr2 (6.32), Vpreb3 (5.87), S100a8 (5.78), Hbb-bt (5.78), Fcrl5 (5.58), Cd22 (5.57), Il5ra (5.36), Iglv3 (5.28), Rgs13 (5.28), Cxcr5 (5.04), Iglc2 (5.01)	CD19+
DC	A530099J19Rik (7.34), Klk1 (7.13), Cldn1 (6.42), Slco5a1 (6.33), Tlr11 (6.05), Adam23 (6.04), Fscn1 (5.98), Xcr1 (5.97), Cacnb3 (5.91), Clec9a (5.85), Mreg (5.84), Siglech (5.78), Strip2 (5.75), Ccl22 (5.67), H2-M2 (5.67), Krt5 (5.44), Slc4a8 (5.11), Cd207 (5.06), Zc3h12c (5.04), Arhgap28 (5.02)	CD11c+
Endothelial Cells	Glycam1 (10.3), Enpp2 (9.8), Mmrn1 (9.3), Plvap (9.14), Cpe (9.01), Tnc (8.8), Sema3d (8.74), Cyr61 (8.68), Cyp1b1 (8.42), Madcam1 (8.37), Mmrn2 (8.28), Timp3 (8.23), Jam3 (8.23), Tm4sf1 (8.21), Dsg2 (8.16), Postn (8.1), Serpina3n (8.02), Sparcl1 (7.99), Nfib (7.98), Cav1 (7.95), Lama4 (7.9), Ramp2 (7.84), Emcn (7.79), Sdpr (7.77), Clca3a1 (7.77), Rnd1 (7.73), Prox1 (7.69), Meox1 (7.68), Fstl1 (7.65), Apold1 (7.62), Serpina1b (7.59), Klhl4 (7.58), Pgm5 (7.53), Gpm6a (7.49), Gja1 (7.49), 8430408G22Rik (7.48), Tie1 (7.44), Rassf9 (7.44), Ackr4 (7.4), Myct1 (7.37), Adgrf5 (7.3), Sema3a (7.26), Tspan12 (7.25), Chst4 (7.23), Ephb4	CD45-

	(7.22), Susd2 (7.16), Adamts1 (7.12), Arhgap42 (7.11), Tspan7 (7.08), Il33 (7.07), Osmr (7.04), Col12a1 (7.02), Meox2 (7.02), Nts (7.01), Rhoj (6.99), Gdf10 (6.97), Timp4 (6.96), Ehd2 (6.93), Casp12 (6.89), Cldn5 (6.88), Arhgap29 (6.85), Tjp1 (6.84), Tek (6.8), Sparc (6.78), Clu (6.77), Sncaip (6.73), Igfbp7 (6.72), Rasip1 (6.71), Podxl (6.7), Ces2g (6.7), Ecscr (6.68), Kdr (6.65), Ptprk (6.65), Lrg1 (6.64), Ecm2 (6.62), AW551984 (6.62), 1810011O10Rik (6.58), Fbn1 (6.52), Ltbp4 (6.49), Egfl7 (6.44), Flt4 (6.4), Ces2e (6.37), Aqp1 (6.35), Parva (6.33), Prelp (6.33), Ldb2 (6.3), Cdh5 (6.28), Edn1 (6.28), Hspg2 (6.28), Fermt2 (6.27), Lamb1 (6.27), Sele (6.27), Galnt18 (6.27), Igfbp3 (6.26), Fmo1 (6.23), Crip2 (6.2), Sox18 (6.19), Ch25h (6.18), Slco2a1 (6.18), Rgs4 (6.14), C1s1 (6.14), Sh3gl3 (6.12), Csf1 (6.11), Hoxd8 (6.11), Me1 (6.1), Flrt2 (6.06), Prss23 (6.05), Plk2 (6.03), Col4a1 (6.02), Stab2 (6.02), Aplnr (6), Akr1c14 (5.98), Fzd6 (5.96), Fkbp10 (5.95), Esam (5.95), Zfp521 (5.94), Ubd (5.93), Rgs16 (5.9), Hsp25-ps1 (5.89), Epas1 (5.88), Fzd4 (5.87), Pxdn (5.84), Fabp4 (5.84), Clec14a (5.82), Nr2f2 (5.82), Plat (5.81), Adamts9 (5.81), Btnl9 (5.81), Mmp15 (5.8), Meis2 (5.79), Bgn (5.78), Nckap1 (5.78), S100a16 (5.77), Cyyr1 (5.76), Mid2 (5.76), Ackr1 (5.74), Plscr2 (5.69), Cp (5.69), Lbp (5.68), Nectin2 (5.68), Phldb2 (5.68), Tmem47 (5.68), F8 (5.68), Dchs1 (5.66), 4930578C19Rik (5.66), Robo4 (5.65), Fkbp9 (5.63), Clec1a (5.63), Vwf (5.63), Ptrf (5.61), Sorbs2 (5.6), Tmem204 (5.57), Ddah1 (5.57), Efna1 (5.56), Pcdh17 (5.56), Col4a2 (5.55), Parm1 (5.55), Pdgfd (5.54), Col15a1 (5.53), Ptprb (5.53), Serping1 (5.53), Nxpe2 (5.53), Tspan18 (5.53), Npr1 (5.52), Plxna2 (5.5), Procr (5.49), Grb10 (5.47), Tspan15 (5.47), Cfb (5.47), Tll1 (5.46), Fbln5 (5.46), A4galt (5.45), Pls3 (5.45), Coch (5.45), Gpr182 (5.43), Lhfp (5.41), Cldn11 (5.4), Shank3 (5.4), Fgd5 (5.4), Jam2 (5.4), Ccl20 (5.39), Fhdc1 (5.38), Prkcdbp (5.38), Bace2 (5.37), Cyp4b1 (5.35), Abcg2 (5.35), Ica1 (5.35), Igfbp5 (5.34), Il13ra2 (5.32), Tinagl1 (5.31), Eng (5.28), Cxcl12 (5.28), Cd300lg (5.27), Adgrl4 (5.27), Upp1 (5.26), Tns2 (5.26), Tgm2 (5.25), Ptprg (5.24), Foxc2 (5.23), Pkhd1l1 (5.23), Dock9 (5.23), Stc1 (5.23), Bcl6b (5.22), Ltbp2 (5.21), Olfr1372-ps1 (5.21), Apbb2 (5.2), Tmem88 (5.2), Nid1 (5.19), C1qtnf7 (5.19), Pam (5.18), Bmp4 (5.15), Ace (5.14), Angpt2 (5.14), Dpysl3 (5.14), Ushbp1 (5.13), Oit1 (5.13), Angptl4 (5.12), 9430020K01Rik (5.1), Vwa1 (5.1), Nos3 (5.1), Bcam (5.09), Myo1b (5.09), Unc5b (5.08), Ifi44 (5.08), Yes1 (5.06), Dll4 (5.06), Wwtr1 (5.06), Npr2 (5.05), Pdpn (5.03), Gxylt2 (5.03), Rgs5 (5.02), Tmtc1 (5.02), Cd34 (5.01), Ptprm (5.01), Npdc1 (5.01)	
Macrophage	Vsig4 (9.95), Atp6v0d2 (9.04), Ear1 (8.72), Cd209f (8.64), Cd5l (8.51), Fabp7 (8.42), C1qc (8.33), Prg4 (8.31), C3ar1 (8.27), Adamdec1 (8.07), Alox15 (8.07), Saa3 (7.86), Clec4n (7.76), Pf4	CD11b+

	(7.66), C1qa (7.62), Fcgr4 (7.59), C6 (7.55), Gpnmb (7.44), Mmp13 (7.43), F5 (7.37), Slc40a1 (7.27), C1qb (7.25), Mertk (7.25), Cd300ld2 (7.13), Gbp2b (7.11), Ms4a4a (7.09), C5ar1 (7.03), Ms4a7 (6.91), Mafb (6.88), Ms4a14 (6.87), Klra2 (6.79), Alb (6.74), Fpr1 (6.73), Folr2 (6.65), Naip1 (6.6), Igha (6.53), Cbr2 (6.51), Ccl8 (6.5), Clec1b (6.48), Mrc1 (6.43), Cxcl13 (6.42), C4b (6.42), Ccl12 (6.41), Tfec (6.4), Adgre1 (6.38), Tlr8 (6.31), Clec4d (6.23), Olr1 (6.21), Pla2g2d (6.19), Hacd4 (6.1), Hal (6.1), Itgb5 (6.09), Cd163 (6.07), Slc11a1 (5.98), Lilra5 (5.95), Agmo (5.94), Chil3 (5.93), Pla2g7 (5.93), Slc7a2 (5.9), Slc7a8 (5.87), Fgd4 (5.85), Ccl24 (5.83), Fcgr1 (5.82), Abcd2 (5.81), Cd300ld (5.8), Ighv8-5 (5.79), Abcc3 (5.79), Msr1 (5.79), Ms4a6d (5.76), Igf1 (5.76), Fcna (5.76), Clec4a3 (5.75), Csf1r (5.71), Ccr1 (5.69), Ccr3 (5.68), Ms4a8a (5.68), Serpinb10 (5.66), Fpr2 (5.65), Hmox1 (5.63), Defa24 (5.63), Ptgs1 (5.62), Cd209g (5.59), Prss34 (5.58), Wfdc17 (5.58), Padi4 (5.55), Axl (5.54), Spic (5.53), Clec7a (5.5), Clec4f (5.46), Pilrb1 (5.45), Abca9 (5.43), Krt19 (5.43), Fabp1 (5.42), Ccl6 (5.41), Fcgr3 (5.4), Plau (5.4), Pilra (5.39), Cd209b (5.38), Reg3g (5.38), Ednrb (5.38), Siglec1 (5.36), Apoe (5.35), Plet1 (5.34), Serpina1a (5.31), Pmp22 (5.3), Lpl (5.28), F10 (5.26), Cd302 (5.26), Tbxas1 (5.25), Spp1 (5.24), Car4 (5.22), Adgb (5.21), Lrp1 (5.2), Clec4a1 (5.2), Hpgds (5.18), Gm5150 (5.16), Hpgd (5.16), Etv1 (5.14), F13a1 (5.14), Mcpt8 (5.13), Ecm1 (5.11), Ifi204 (5.09), Dmxl2 (5.06), Gda (5.05), Igsf6 (5.04)	
NK	Klra8 (11.49), Klra7 (9.89), Ncr1 (9.59), Klra13-ps (8.15), NA.94 (8.08), Klri2 (8.04), Klrb1a (7.87), Klre1 (7.44), Klra6 (7.18), Klrb1c (7.02), Klrc3 (6.77), Klra4 (6.45), Adamts14 (6.3), Khdc1a (6.27), Itga2 (6.17), Cma1 (5.99), Styk1 (5.76), Klra1 (5.68), Samd3 (5.6), Klrc2 (5.56), Klra3 (5.55), Gzma (5.55), Prf1 (5.39), Eomes (5.13), Spry2 (5.12), Klra10 (5.05)	NK1.1
T cells	Trac (8.6), Themis (8.35), Traj49 (7.54), Cd3g (7.52), Trav14d-3-dv8 (7.15), Dapl1 (7.09), Cd3e (6.95), Cd3d (6.68), Trav7-5 (6.56), Trav15-1-dv6-1 (6.18), Trav14-1 (6), Traj41 (5.99), Cd5 (5.96), Trat1 (5.88), Cd6 (5.86), Lat (5.67), Traj44 (5.34), Aqp11 (5.25), Trbv29 (5.15), Cd28 (5.11)	CD4+ or CD8+

Supplementary Table 5. The gene sets and CITE-seq markers used for cell typing of the B16F10 mouse model of melanoma. For each gene, the value following it in parentheses is its gene weight. The CITE-seq markers selected reflect canonical markers of cell types (2).

Cell Type	Genes	CITE-seq
Macrophage	GPNMB (12.52), CHI3L1 (12.48), CCL18 (12.3), MRC1 (11.9), PLA2G7 (11.69), FABP4 (11.35), MMP7 (11.26), LPL (11.12),	CD14+

	CXCL5 (11.11), SPP1 (11.04), C1QB (10.86), C1QC (10.54), SEPP1 (10.47), TNFAIP6 (10.41), CHIT1 (10.24), C1QA (10.21), MS4A4A (10.13), ADAMDEC1 (10.03), GREM1 (9.96), APOC1 (9.96), SERPINB2 (9.91), APOE (9.88), CD163 (9.77), DCSTAMP (9.73), A2M (9.72), CCL2 (9.71), RNASE1 (9.69), FBP1 (9.66), IDO1 (9.52), SDC2 (9.27), ANPEP (9.25), GJB2 (9.21), EMP1 (9.18), CXCL3 (9.16), CYP1B1 (9.13), TMIGD3 (9.09), DOCK4 (9.08), PMP22 (9.06), MMP12 (9.06), MMP9 (9.05), C5AR1 (8.96), AQP9 (8.95), MYOF (8.94), CCL8 (8.92), MMP1 (8.91), NR1H3 (8.75), HS3ST2 (8.7), RIN2 (8.67), F13A1 (8.67), ALDH1A1 (8.65), CCL22 (8.63), GLDN (8.63), NCEH1 (8.61), VSIG4 (8.56), IGSF6 (8.54), CXCL10 (8.52), LILRB4 (8.49), SUCNR1 (8.49), SHTN1 (8.42), C15orf48 (8.42), SLAMF8 (8.39), SCG5 (8.36), FPR3 (8.34), IL1RN (8.32), CLEC5A (8.31), CYP27B1 (8.28), GPX3 (8.15), PTGS2 (8.11), MAFB (8.06), CXCL1 (8.06), IL1A (8.02), PLAUR (8.01), CCL23 (8.01), OLR1 (8), GPC4 (7.94), SPARC (7.92), INHBA (7.91), SLC38A6 (7.88), EPB41L3 (7.87), TFEC (7.87), NCF2 (7.83), PLPP3 (7.82), DAB2 (7.81), CXCL2 (7.8), SERPING1 (7.79), F3 (7.77), TIMP2 (7.76), CXCL16 (7.74), MGST1 (7.74), TNFSF15 (7.73), NPL (7.71), ADGRE2 (7.68), CD68 (7.64), EREG (7.59), DRAM1 (7.57), CD36 (7.54), CXCL8 (7.49), PTX3 (7.49), GAL (7.49), NONAME405 (7.48), NDP (7.46), CXCL9 (7.45), HAMP (7.42), PSD3 (7.36), NRP1 (7.36), TREM2 (7.32), CCL13 (7.31), HK3 (7.3), ACOD1 (7.3), C11orf45 (7.28), HSD11B1 (7.28), CLEC10A (7.27), PPARG (7.24), RAB42 (7.23), PILRA (7.18), DNASE2B (7.17), TUBB6 (7.08), CTSK (7.06), CLIC2 (7.04), ELOVL7 (7.02), CCRL2 (7.01), CREG1 (6.99), CA2 (6.99), PTGR1 (6.98), CEBPA (6.97), PLTP (6.95), LGMN (6.95), HTRA4 (6.94), HMOX1 (6.94), GDF15 (6.94), PDGFC (6.91), CYFIP1 (6.9), SLC7A11 (6.9), MS4A7 (6.88), ADAM9 (6.87), FAM198B (6.85), CTSB (6.83), PCOLCE2 (6.82), CXCL11 (6.82), ZC3H12C (6.81), IL13RA1 (6.81), PLXDC2 (6.81), GPR84 (6.8), TLR8 (6.77), MSR1 (6.77), HCK (6.74), IFI27 (6.73), OLFML2B (6.73), ARRDC4 (6.71), ACP5 (6.7), GM2A (6.7), RTN1 (6.68), MCTP1 (6.68), DFNA5 (6.67), FOXQ1 (6.66), CNIH3 (6.62), SLC47A1 (6.6), CYP27A1 (6.59), OSCAR (6.58), CCL26 (6.58), PLD3 (6.57), CRABP2 (6.55), CPM (6.54), COL8A2 (6.52), CD14 (6.52), SLC28A3 (6.52), C3 (6.51), MGLL (6.5), SRPX (6.49), TGFBI (6.44), SIRPA (6.42), ADORA3 (6.41), DMXL2 (6.39), GGTA1P (6.38), CPVL (6.37), ZMIZ1-AS1 (6.36), CD300LF (6.35), PLA2G4A (6.34), MREG (6.34), TMEM176B (6.33), TTC7B (6.31), TREM1 (6.31), MARCKS (6.31), G0S2 (6.29), RASSF4 (6.29), SERPINE1 (6.27), TNIP3 (6.26), CTSD (6.26), MS4A6E (6.26), CCL20 (6.25), GAS2L3 (6.25), OSBPL1A (6.25), LILRB2 (6.24), IL1B (6.24), CCND1 (6.22), VMO1 (6.2), ATP1B1	

	(6.2), SLC31A2 (6.2), CSF2RA (6.18), TIMP3 (6.16), IGFBP6 (6.15), RARRES1 (6.14), FMNL2 (6.14), TGM2 (6.14), ME1 (6.14), ACSL1 (6.13), CAPG (6.11), MERTK (6.11), RNF130 (6.09), TIE1 (6.09), MARCO (6.08), RGL1 (6.08), RAI14 (6.07), SCARB2 (6.07), CTSL (6.07), CLEC7A (6.06), PHLDA2 (6.05), CCL7 (6.05), HSD3B7 (6.05), SLC15A3 (6.04), CSF1R (6.04), CFD (6.02), CD9 (6.01), SIGLEC1 (5.96), FADS1 (5.95), FCHO2 (5.94), TLR2 (5.93), NRIP3 (5.92), MT1M (5.91), NUPR1 (5.91), FAM213A (5.9), KCTD12 (5.88), EPHX1 (5.85), HTRA1 (5.85), CFB (5.85), SOD2 (5.84), PAPSS2 (5.84), TMEM255A (5.83), PLBD1 (5.83), GEM (5.8), GSN (5.8), HPGDS (5.79), RBP4 (5.79), SDC4 (5.78), EMILIN2 (5.77), ABCG2 (5.76), TNS3 (5.75), ACP2 (5.75), GRN (5.75), SPOCD1 (5.73), RAB7B (5.72), SLC16A3 (5.7), MCOLN3 (5.7), ITGAX (5.68), VNN1 (5.67), CSRP2 (5.67), FUCA1 (5.65), TM4SF1 (5.64), LRP12 (5.64), LILRA2 (5.64), KCNJ2 (5.63), TLR4 (5.63), THBS1 (5.62), LILRA6 (5.62), TYMP (5.61), P2RX7 (5.6), CCR1 (5.6), MRAS (5.59), DHRS11 (5.59), SLC7A7 (5.59), MAOA (5.59), CORO1C (5.58), IL17RB (5.58), CD274 (5.58), METTL7B (5.56), SLC29A3 (5.52), TFPI2 (5.5), LACC1 (5.49), CLDN23 (5.49), GNS (5.47), RAMP1 (5.47), LINC01010 (5.45), TCEAL9 (5.44), RBM47 (5.44), RND3 (5.43), ALOX15B (5.43), IL12B (5.43), RAB20 (5.42), PALLD (5.4), SPHK1 (5.39), CDKN1A (5.39), ASAP2 (5.39), SEMA3C (5.37), LILRB5 (5.37), APOBEC3A (5.36), SLC6A12 (5.35), SPRY2 (5.33), TACSTD2 (5.31), PGD (5.29), LINC01503 (5.28), GAPLINC (5.27), RNF128 (5.26), MT1G (5.26), NME7 (5.25), IL18 (5.25), MUCL1 (5.24), ATP6V0D2 (5.23), ANKRD22 (5.22), IRAK3 (5.22), NONAME190 (5.2), KYNU (5.2), BHLHE41 (5.2), ABCA1 (5.19), LPCAT2 (5.18), FCGRT (5.17), RAB31 (5.17), IL1R2 (5.17), LINC01094 (5.16), PYGL (5.16), LGALS3 (5.15), P2RY13 (5.15), CTSZ (5.14), PPIC (5.13), CYBRD1 (5.12), SMPDL3A (5.12), MIR3945HG (5.12), MMP10 (5.12), PEA15 (5.12), LDLRAD3 (5.12), HBEGF (5.11), SULF2 (5.11), SLC11A1 (5.11), TDRD9 (5.11), FTH1 (5.1), BLVRB (5.1), SEPT10 (5.1), IL4I1 (5.1), MS4A6A (5.09), EMILIN1 (5.09), DSE (5.08), IL10RB-AS1 (5.07), IDH1 (5.06), TNFRSF21 (5.06), FGR (5.06), ICAM1 (5.05), TTYH3 (5.04), ACO1 (5.03), ALDH1A2 (5.03), SLC37A2 (5.03), TBC1D8 (5.02), ALAS1 (5.02), LIPA (5.01), PLAU (5.01)	
NK cells	SH2D1B (8.91), CLIC3 (7.38), XCL1 (6.78), KIT (6.72), KIR3DL1 (6.46), KIR2DL4 (6.27), FASLG (5.79), KLRF1 (5.78), KIR3DL2 (5.73), NR4A2 (5.71), PRR5L (5.68), HOXA5 (5.68), KRT86 (5.58), YES1 (5.43), S1PR5 (5.42), IL18RAP (5.41), SLFN13 (5.35), IGFBP7 (5.3), AREG (5.26), PTGDR (5.08), MTRF1L (5.06)	CD56+

T cells	TRAT1 (9.15), THEMIS (8.53), MAL (8.35), CD3G (8.25), CD8B (8.2), LRRN3 (7.7), NELL2 (7.55), CD3D (7.35), TRAJ41 (6.63), INPP4B (6.48), CD8A (6.43), TCF7 (6.43), TRAV13-1 (6.2), GPR171 (6.16), BCL11B (6.11), NOG (6.09), EGFL6 (6.08), TRAC (5.87), LEF1 (5.79), UBASH3A (5.78), KLRG1 (5.73), TC2N (5.67), ICOS (5.63), GZMK (5.53), SIRPG (5.53), CD3E (5.53), RNF157 (5.42), ITK (5.4), CD2 (5.4), TRBC1 (5.36), ANXA3 (5.25), TRAV13-2 (5.23), IPCEF1 (5.23), FLT3LG (5.16), LINC01619 (5.15), GIMAP7 (5.11), TRAV12-3 (5.1), TRAV12-1 (5.09), TRABD2A (5.05), RASGRF2 (5.05), ITM2A (5.01)	CD4+ or CD8+

Supplementary Table 6. The gene sets and CITE-seq markers used for cell typing of the MALT and lymphoma data. The lymphoma data does not have CITE-seq, so the CITE-seq marker column only applies to the MALT dataset. For each gene, the value following it in parentheses is its gene weight. The CITE-seq markers selected reflect canonical markers of cell types (1, 2).

Cell types	Genes
B cells	Ms4a1 (9.46), Cr2 (8.74), Cd19 (8.28), Pax5 (8.09), Fcer2a (8.05), Fcmr (7.85), Cd79a (7.64), Ebf1 (7.25), Ighd (7.2), Pou2af1 (6.72), Blk (6.71), Chst3 (6.5), Cd79b (6.5), Scd1 (6.45), Cecr2 (6.32), Vpreb3 (5.87), S100a8 (5.78), Hbb-bt (5.78), Fcrl5 (5.58), Cd22 (5.57), Il5ra (5.36), Iglv3 (5.28), Rgs13 (5.28), Cxcr5 (5.04), Iglc2 (5.01), Igkv2-109 (5), Igkv1-135 (4.87), Igkv19-93 (4.85), Dennd5b (4.85), S100a9 (4.85), Fcrl1 (4.81), Aicda (4.7), Bank1 (4.63), Igll1 (4.62), Spib (4.6), Igkv12-46 (4.59), Ighv13-2 (4.57), Tnfrsf13c (4.55), Retnlg (4.54), Ralgps2 (4.52), Ltf (4.49), Fam129c (4.48), Vpreb1 (4.47), Hbb-bs (4.46), Akap12 (4.43), Pik3c2b (4.41), Klhl14 (4.4), Ngp (4.33), Fcrla (4.3), Siglecg (4.26), Blnk (4.25), Ighg3 (4.24), Angptl1 (4.22), B3gnt5 (4.19), A530040E14Rik (4.16), Bhlhe41 (4.07), Mybl1 (4.05), Mzb1 (3.95), Gm32819 (3.94), Susd1 (3.88), Ighv1-67 (3.81), Igkv1-117 (3.8), Ighv8-8 (3.77), Cacna1i (3.74), H2-Ob (3.74), Igkv5-45 (3.73), Igkv12-44 (3.72), Pgap1 (3.67), Mmp8 (3.65), Ffar1 (3.64), Il12a (3.64), Lcn2 (3.59), Nuggc (3.56), Igkv6-17 (3.55), Ccr6 (3.55), Mef2c (3.55), Myl4 (3.54), Bfsp2 (3.54), 1810046K07Rik (3.54), Igkv9-120 (3.5), Ighv1-55 (3.47), Cxcr2 (3.45), Gga2 (3.45), Rdh12 (3.44), Bcar3 (3.44), Fbxw13 (3.43), Iglv2 (3.38), Ly6g (3.37), Lgr5 (3.32), Srpk3 (3.31), Snn (3.31), Prkce (3.27), Myo1e (3.27), Cd55 (3.26), Ighv1-42 (3.22), Bcl7a (3.2), Diras2 (3.18), Ighg2b (3.17), Gm9861 (3.15), Ighm (3.14), Bcl11a (3.13), Igkv14-111 (3.1), Gpam (3.09), Ighv1-82 (3.07), Cnr2 (3.06), Syk (3.03), Ighg1 (3.02), Pkig (3.01)
DC	A530099J19Rik (7.34), Klk1 (7.13), Cldn1 (6.42), Slco5a1 (6.33), Tlr11 (6.05), Adam23 (6.04), Fscn1 (5.98), Xcr1 (5.97), Cacnb3 (5.91), Clec9a (5.85), Mreg (5.84), Siglech (5.78), Strip2 (5.75), Ccl22 (5.67), H2-M2 (5.67), Krt5 (5.44), Slc4a8 (5.11), Cd207 (5.06), Zc3h12c (5.04), Arhgap28 (5.02), Il12b (4.83), Apol7c (4.77), Tbc1d8 (4.74), Sftpc (4.62), Clec4a4

	(4.6), Plxnc1 (4.5), Mmp25 (4.5), Nudt17 (4.47), Sept3 (4.46), Naalad2 (4.46), Gpr33 (4.44), Flt3 (4.4), Ccl17 (4.38), Fmnl2 (4.36), Asprv1 (4.34), Krt14 (4.33), Cd300c (4.32), Mab21l3 (4.19), Klri1 (4.19), Tbc1d4 (4.16), Pfkfb3 (4.16), Slc22a23 (4.16), Itgb8 (4.15), Ifi205 (4.13), Tmem150c (4.13), Slc46a3 (4.1), Adam8 (4.02), Hmgn3 (3.96), Ccdc88a (3.94), Mx1 (3.92), Il15 (3.91), Gcsam (3.86), Hepacam2 (3.85), Klra17 (3.83), Il6 (3.83), Ttc39a (3.82), Rogdi (3.8), Tlr3 (3.78), Aldh1a2 (3.77), Dscam (3.77), Bcl2l14 (3.77), Cdk14 (3.75), Clec4b2 (3.71), Nectin1 (3.7), Grk3 (3.68), Havcr1 (3.67), F630111L10Rik (3.66), Pak1 (3.66), Arhgef40 (3.62), Gca (3.6), Fnbp1l (3.6), Zfp366 (3.6), Cxx1c (3.59), Adam11 (3.57), Tbc1d9 (3.57), Arpin (3.55), Tspan33 (3.54), Eno3 (3.51), Gpr157 (3.5), Nostrin (3.47), Papss2 (3.45), Eno2 (3.43), Cyp4f16 (3.42), F830045P16Rik (3.41), Rnd3 (3.41), Tnip3 (3.4), Ogfrl1 (3.38), Ndnf (3.38), Gm11545 (3.36), Pmaip1 (3.35), Kmo (3.35), Adcy6 (3.34), Plbd1 (3.34), Net1 (3.34), Socs2 (3.34), Arc (3.33), Il15ra (3.31), Cd209a (3.3), Nek6 (3.3), Olfr164 (3.3), Tm4sf5 (3.29), 1700009J07Rik (3.26), Ffar4 (3.24), Samsn1 (3.24), Anxa3 (3.22), Mycl (3.22), Slc41a2 (3.21), Cxcl16 (3.21), Insm1 (3.2), Zfp872 (3.18), Tmtc2 (3.17), Relb (3.17), Naaa (3.14), Fam149a (3.14), Snx22 (3.13), Ube2l6 (3.13), Cdh2 (3.13), Smim5 (3.12), Slc7a11 (3.11), Lrp8 (3.11), Lad1 (3.11), Adgrg6 (3.09), Poglut1 (3.09), Cd209e (3.09), Pik3cb (3.08), H2-Eb2 (3.06), Prss30 (3.06), Dsc3 (3.05), Qpct (3.05), Gpr82 (3.04), Cdh1 (3.04), Bmp2k (3.03), Grm8 (3.02), Dcstamp (3.02), Gm15698 (3.01), Scn3a (3), Shtn1 (3), Cxcl15 (3), Plekhn1 (3)
Endothelial Cells	Glycam1 (10.3), Enpp2 (9.8), Mmrn1 (9.3), Plvap (9.14), Cpe (9.01), Tnc (8.8), Sema3d (8.74), Cyr61 (8.68), Cyp1b1 (8.42), Madcam1 (8.37), Mmrn2 (8.28), Timp3 (8.23), Jam3 (8.23), Tm4sf1 (8.21), Dsg2 (8.16), Postn (8.1), Serpina3n (8.02), Sparcl1 (7.99), Nfib (7.98), Cav1 (7.95), Lama4 (7.9), Ramp2 (7.84), Emcn (7.79), Sdpr (7.77), Clca3a1 (7.77), Rnd1 (7.73), Prox1 (7.69), Meox1 (7.68), Fstl1 (7.65), Apold1 (7.62), Serpina1b (7.59), Klhl4 (7.58), Pgm5 (7.53), Gpm6a (7.49), Gja1 (7.49), 8430408G22Rik (7.48), Tie1 (7.44), Rassf9 (7.44), Ackr4 (7.4), Myct1 (7.37), Adgrf5 (7.3), Sema3a (7.26), Tspan12 (7.25), Chst4 (7.23), Ephb4 (7.22), Susd2 (7.16), Adamts1 (7.12), Arhgap42 (7.11), Tspan7 (7.08), Il33 (7.07), Osmr (7.04), Col12a1 (7.02), Meox2 (7.02), Nts (7.01), Rhoj (6.99), Gdf10 (6.97), Timp4 (6.96), Ehd2 (6.93), Casp12 (6.89), Cldn5 (6.88), Arhgap29 (6.85), Tjp1 (6.84), Tek (6.8), Sparc (6.78), Clu (6.77), Sncaip (6.73), Igfbp7 (6.72), Rasip1 (6.71), Podxl (6.7), Ces2g (6.7), Ecscr (6.68), Kdr (6.65), Ptprk (6.65), Lrg1 (6.64), Ecm2 (6.62), AW551984 (6.62), 1810011O10Rik (6.58), Fbn1 (6.52), Ltbp4 (6.49), Egfl7 (6.44), Flt4 (6.4), Ces2e (6.37), Aqp1 (6.35), Parva (6.33), Prelp (6.33), Ldb2 (6.3), Cdh5 (6.28), Edn1 (6.28), Hspg2 (6.28), Fermt2 (6.27), Lamb1 (6.27), Sele (6.27), Galnt18 (6.27), Igfbp3 (6.26), Fmo1 (6.23), Crip2 (6.2), Sox18 (6.19), Ch25h (6.18), Slco2a1 (6.18), Rgs4 (6.14), C1s1 (6.14), Sh3gl3 (6.12), Csf1 (6.11), Hoxd8 (6.11), Me1 (6.1), Flrt2 (6.06), Prss23 (6.05), Plk2 (6.03), Col4a1 (6.02), Stab2 (6.02), Aplnr (6), Akr1c14 (5.98), Fzd6 (5.96), Fkbp10 (5.95), Esam (5.95), Zfp521 (5.94), Ubd (5.93), Rgs16

	(5.9), Hsp25-ps1 (5.89), Epas1 (5.88), Fzd4 (5.87), Pxdn (5.84), Fabp4 (5.84), Clec14a (5.82), Nr2f2 (5.82), Plat (5.81), Adamts9 (5.81), Btnl9 (5.81), Mmp15 (5.8), Meis2 (5.79), Bgn (5.78), Nckap1 (5.78), S100a16 (5.77), Cyyr1 (5.76), Mid2 (5.76), Ackr1 (5.74), Plscr2 (5.69), Cp (5.69), Lbp (5.68), Nectin2 (5.68), Phldb2 (5.68), Tmem47 (5.68), F8 (5.68), Dchs1 (5.66), 4930578C19Rik (5.66), Robo4 (5.65), Fkbp9 (5.63), Clec1a (5.63), Vwf (5.63), Ptrf (5.61), Sorbs2 (5.6), Tmem204 (5.57), Ddah1 (5.57), Efna1 (5.56), Pcdh17 (5.56), Col4a2 (5.55), Parm1 (5.55), Pdgfd (5.54), Col15a1 (5.53), Ptprb (5.53), Serping1 (5.53), Nxpe2 (5.53), Tspan18 (5.53), Npr1 (5.52), Plxna2 (5.5), Procr (5.49), Grb10 (5.47), Tspan15 (5.47), Cfb (5.47), Tll1 (5.46), Fbln5 (5.46), A4galt (5.45), Pls3 (5.45), Coch (5.45), Gpr182 (5.43), Lhfp (5.41), Cldn11 (5.4), Shank3 (5.4), Fgd5 (5.4), Jam2 (5.4), Ccl20 (5.39), Fhdc1 (5.38), Prkcdbp (5.38), Bace2 (5.37), Cyp4b1 (5.35), Abcg2 (5.35), Ica1 (5.35), Igfbp5 (5.34), Il13ra2 (5.32), Tinagl1 (5.31), Eng (5.28), Cxcl12 (5.28), Cd300lg (5.27), Adgrl4 (5.27), Upp1 (5.26), Tns2 (5.26), Tgm2 (5.25), Ptprg (5.24), Foxc2 (5.23), Pkhd1l1 (5.23), Dock9 (5.23), Stc1 (5.23), Bcl6b (5.22), Ltbp2 (5.21), Olfr1372-ps1 (5.21), Apbb2 (5.2), Tmem88 (5.2), Nid1 (5.19), C1qtnf7 (5.19), Pam (5.18), Bmp4 (5.15), Ace (5.14), Angpt2 (5.14), Dpysl3 (5.14), Ushbp1 (5.13), Oit1 (5.13), Angptl4 (5.12), 9430020K01Rik (5.1), Vwa1 (5.1), Nos3 (5.1), Bcam (5.09), Myo1b (5.09), Unc5b (5.08), Ifi44 (5.08), Yes1 (5.06), Dll4 (5.06), Wwtr1 (5.06), Npr2 (5.05), Pdpn (5.03), Gxylt2 (5.03), Rgs5 (5.02), Tmtc1 (5.02), Cd34 (5.01), Ptprm (5.01), Npdc1 (5.01), Lamc1 (4.98), Spns2 (4.98), Kitl (4.97), AU021092 (4.97), Flt1 (4.97), Lmcd1 (4.96), Itga1 (4.96), Thbd (4.96), Foxp2 (4.95), Adcy4 (4.94), Tmem252 (4.93), Slc10a6 (4.93), Myzap (4.92), Pkn3 (4.92), Sema5a (4.92), C1ql3 (4.92), Ctnnal1 (4.91), Palmd (4.91), Gja4 (4.9), Hamp (4.89), Lamb2 (4.89), Bmp2 (4.88), Svs2 (4.88), Plpp3 (4.88), Tnfsf10 (4.88), Tmem2 (4.88), Efnb2 (4.87), F2r (4.86), Il1r1 (4.85), Galnt15 (4.85), Lyve1 (4.85), Ppic (4.84), Zfp979 (4.84), C130074G19Rik (4.83), Ackr2 (4.83), Kcnj2 (4.83), Serpine1 (4.83), Cav2 (4.82), Ier3 (4.82), C3 (4.8), B3gnt3 (4.8), Plcd1 (4.8), Plpp1 (4.79), Fxyd6 (4.79), Pcdh7 (4.79), Atp8b1 (4.77), Rapgef5 (4.77), Cdc42ep1 (4.76), Sema3f (4.75), Arhgef15 (4.74), Tgfb3 (4.73), Eln (4.72), Sulf1 (4.72), Etl4 (4.71), Olfml2a (4.7), Gng11 (4.7), Pawr (4.69), Fbxl7 (4.68), Serpinh1 (4.67), Agrn (4.67), Itga2b (4.67), Sox17 (4.67), Lama5 (4.66), Armcx1 (4.66), Serpina3f (4.66), Dcn (4.66), Fam198b (4.64), Btbd3 (4.64), Pald1 (4.64), Nqo1 (4.63), Syt15 (4.63), Sema6a (4.63), Popdc2 (4.63), Rbp1 (4.62), Lrrc32 (4.62), Rcan1 (4.61), Cdh13 (4.61), Mustn1 (4.61), Prex2 (4.61), Sox7 (4.6), F11r (4.59), Chrm3 (4.59), Nudt4 (4.58), Raet1e (4.58), Kcng3 (4.58), Fmo2 (4.57), Stap2 (4.57), Mgll (4.57), Arrdc4 (4.56), Tnfrsf10b (4.56), Ptpn14 (4.56), Tgfbr3 (4.55), Ghr (4.55), Hspa12b (4.54), Lims2 (4.54), Iigp1 (4.54), Cd151 (4.54), Lhfpl2 (4.53), Adcy5 (4.52), Colgalt2 (4.52), Nov (4.52), Hyal1 (4.51), Fat4 (4.51), Itih5 (4.5), Ctla2a (4.49), Cdc42bpa (4.48), Prnp (4.46), Tgfa (4.46), Hoxd10 (4.46), Arap3 (4.46), Chst2 (4.45), Bcar1 (4.45), Serpine2 (4.44), Fbln2 (4.43), Atp9a (4.42), Arhgap23 (4.41), Shroom4 (4.39), Clic5 (4.38), Tmem98 (4.37), Itga3 (4.36), C2 (4.36), Cdc42ep5 (4.35), Ndn (4.35), Lix1l

	(4.33), Sema7a (4.33), Aqp7 (4.33), Slc9a3r2 (4.31), Car8 (4.31), Msrb3 (4.31), Plcb4 (4.31), Erg (4.29), Bmx (4.29), Scarf1 (4.28), Svs4 (4.27), Adamts5 (4.27), Aebp1 (4.27), Il7 (4.27), Heph (4.26), Fam167b (4.26), Esm1 (4.26), Arhgef10 (4.25), Plekhg1 (4.25), Mcam (4.24), Cyp2j6 (4.24), Tbx1 (4.24), Smtn (4.23), Mfge8 (4.22), Tfpi (4.21), Abi3bp (4.19), Dll1 (4.19), Foxf1 (4.19), Flrt3 (4.19), Cttn (4.18), Socs3 (4.18), Pcp4l1 (4.17), Cpd (4.17), Slc45a3 (4.16), Maged1 (4.16), Fibin (4.16), Svs5 (4.15), Astn1 (4.15), Fam189a2 (4.15), Phf11d (4.15), Marco (4.13), Fam43a (4.13), Nxn (4.12), Tspan6 (4.12), Ccdc3 (4.11), Ablim3 (4.11), Psg22 (4.11), C1qtnf9 (4.1), Creb3l2 (4.09), Asah2 (4.09), Kank3 (4.09), Pecam1 (4.09), Serpina3i (4.08), Scarb1 (4.08), Ebf3 (4.08), Csrp2 (4.08), Dcbld1 (4.07), Ccdc85a (4.07), BC023105 (4.07), Rdh10 (4.06), Mme (4.06), Dysf (4.06), Gucy1a3 (4.06), Nid2 (4.05), Ppfibp1 (4.05), Id1 (4.05), Spry4 (4.05), Dock6 (4.04), Pcdhb16 (4.04), Enpp3 (4.02), Lrrc3b (4.02), Csgalnact1 (4.02), Armcx4 (4.02), Sept10 (4.01), Robo1 (4), Slit2 (3.99), Crim1 (3.99), Nbea (3.98), Slc16a9 (3.97), Acacb (3.96), Itgb4 (3.95), Ccm2l (3.95), Kank1 (3.94), Tsc22d1 (3.94), Yap1 (3.94), Cd200 (3.93), Slc43a3 (3.93), Mpzl1 (3.93), Mfsd4a (3.93), St6galnac3 (3.92), Tspan4 (3.91), Thsd4 (3.91), Nfia (3.9), Eml1 (3.9), Gem (3.9), Tspan9 (3.9), Plxna4 (3.89), Smad1 (3.88), Dpyd (3.86), Hbegf (3.86), Rcn3 (3.85), Ttll7 (3.85), Ndrg1 (3.85), Mansc1 (3.84), Arsa (3.83), Stra6 (3.81), Ackr3 (3.81), Pkia (3.81), Trim47 (3.81), Mecom (3.79), Cpne8 (3.79), Jag1 (3.78), Caskin2 (3.78), Fam171a1 (3.78), Mageh1 (3.78), Nxph1 (3.77), Mxra8 (3.77), Sema4c (3.77), Efhd1 (3.77), Raet1d (3.76), Nxpe4 (3.76), Mpdz (3.76), Pard6g (3.76), Pik3r3 (3.75), Sncg (3.74), Met (3.74), Myo6 (3.73), Proser2 (3.73), Mcc (3.73), Prkd1 (3.72), Stox2 (3.72), Ctgf (3.72), Arhgef12 (3.72), Cd36 (3.71), Errfi1 (3.71), Lepr (3.71), Gpihbp1 (3.7), Fam13c (3.7), Zfp423 (3.7), Acer2 (3.69), Uaca (3.69), Rai14 (3.69), Slc16a12 (3.68), Pdgfb (3.68), Gadd45g (3.68), Rbfox2 (3.68), C1ra (3.67), Pdk4 (3.67), Stab1 (3.67), She (3.66), Reln (3.66), Samd12 (3.66), Sema3g (3.66), Copz2 (3.65), Ddr2 (3.65), Tril (3.65), Myo1d (3.65), Fkbp7 (3.65), Creg2 (3.64), Sema6b (3.64), Pkd2 (3.64), Piezo2 (3.63), Dnm3 (3.63), Gprc5b (3.62), Lrrn1 (3.62), Amotl1 (3.62), Smagp (3.62), Laptm4b (3.62), Pde7b (3.62), Hyal2 (3.61), Lifr (3.6), Map1b (3.6), Itga5 (3.6), Reck (3.6), Calcrl (3.59), Magi1 (3.59), Dram1 (3.59), Timp1 (3.58), Acvr1 (3.58), Ajuba (3.58), Phyhd1 (3.57), Afdn (3.56), Trim2 (3.56), Dcbld2 (3.56), Ar (3.55), Nrep (3.55), Slco2b1 (3.55), Nos2 (3.54), Cyb561 (3.54), Abcb1a (3.54), Kdelc2 (3.54), Alox12 (3.54), Nr5a2 (3.53), Fos (3.53), Csf2rb2 (3.53), Zcchc14 (3.53), Tmem100 (3.52), Ripply3 (3.52), Dock1 (3.52), Pcdh12 (3.51), Spry1 (3.51), Pcdh1 (3.5), Dusp3 (3.5), Fut7 (3.5), Thbs1 (3.5), Tmem255b (3.5), Eda2r (3.49), Gm6705 (3.49), Tcf7l1 (3.47), Pcdhgb2 (3.47), Amotl2 (3.46), Tead1 (3.46), B4galt4 (3.46), Dok4 (3.45), Gcnt1 (3.45), Klf4 (3.45), Thrsp (3.44), Pear1 (3.44), Gata6 (3.44), Wwc2 (3.44), Klrb1f (3.44), Adm (3.43), Cadps2 (3.43), Unc13b (3.42), Gm9294 (3.42), Plcb1 (3.42), Cxcl1 (3.42), Cd59a (3.41), Prrg1 (3.41), Afap1l1 (3.4), Ece1 (3.4), Slc6a9 (3.39), Ccnd1 (3.39), Ralgds (3.39), Maoa (3.39), Nynrin (3.38), Vangl1 (3.38), Dlc1 (3.38), Mamstr (3.37), Rbp7 (3.37), Jun (3.35), Efr3b

	(3.35), Lgr4 (3.35), Abca1 (3.35), Pcdhb17 (3.34), Kctd12b (3.34), Rapgef3 (3.34), Cdc42bpb (3.34), Mtmr11 (3.33), Rasd1 (3.33), Fam57a (3.33), Ctla2b (3.32), Hs3st1 (3.31), Hmgcs2 (3.31), Reep1 (3.3), Gata2 (3.3), Lama3 (3.3), Dkk2 (3.3), Adgrl2 (3.3), Mical2 (3.3), H1f0 (3.3), Mlkl (3.29), Trip10 (3.29), Notch4 (3.29), Tnfrsf23 (3.29), Slc39a4 (3.29), Acvrl1 (3.28), Nupr1 (3.28), C1qtnf6 (3.28), Oaz2 (3.28), Rassf6 (3.28), App (3.27), Cald1 (3.27), Ttc9 (3.27), Ctsl (3.27), Ephx2 (3.27), Gja5 (3.26), Hsd3b7 (3.26), Scn1b (3.26), Nrp2 (3.26), Smyd2 (3.25), Stxbp1 (3.25), Pim3 (3.25), Pdzd2 (3.24), Fam174b (3.24), Sntb2 (3.24), Ifitm3 (3.24), Klhl29 (3.23), Ralb (3.23), Hoxd4 (3.23), Hey1 (3.23), Lipg (3.23), Tnfaip1 (3.22), Fzd8 (3.22), Hmcn1 (3.22), Arrb1 (3.21), Cx3cl1 (3.21), Vcl (3.2), Rhoc (3.2), Ctnnd1 (3.2), Kirrel (3.2), Nsg1 (3.2), Zfp46 (3.2), Tceal1 (3.19), Rbpms (3.19), Chrnb1 (3.19), Ccdc80 (3.18), Thsd7a (3.18), Dapk2 (3.18), Itga9 (3.18), Gm4951 (3.17), Fam114a1 (3.17), Cebpd (3.17), Clstn1 (3.16), Tcaf1 (3.16), Gab1 (3.16), Pcdhga3 (3.15), Cda (3.14), Zfyve9 (3.14), Egr1 (3.14), Lamc2 (3.13), Nes (3.12), Tagln (3.12), Olfr1396 (3.11), Igsf10 (3.11), Tanc1 (3.11), Pcdhb7 (3.11), Exoc3l2 (3.11), Mapk12 (3.11), Efnb1 (3.1), Psen2 (3.1), Filip1 (3.1), Flnc (3.1), Rras (3.1), Stmn2 (3.1), Plod1 (3.09), Slc18a1 (3.09), Glis3 (3.09), Fosb (3.09), Dclk1 (3.09), Aplp2 (3.09), Pdlim1 (3.08), Adamts4 (3.08), 2310067E19Rik (3.08), Mest (3.08), Gng12 (3.07), Acy3 (3.07), Ccdc141 (3.07), Mmp14 (3.06), Chst1 (3.06), Cdc42ep2 (3.06), Tgfb1i1 (3.05), Vash1 (3.05), Nectin3 (3.05), Doc2b (3.05), Tshz2 (3.05), 4931406P16Rik (3.04), Hoxd9 (3.04), Noct (3.04), Zfp462 (3.04), Tfpi2 (3.04), Epha2 (3.03), Wscd1 (3.03), Grrp1 (3.02), Cyp2d22 (3.02), Atf3 (3.02), Serpina3g (3.02), Limch1 (3.01), Myo10 (3.01)
Macrophage	Vsig4 (9.95), Atp6v0d2 (9.04), Ear1 (8.72), Cd209f (8.64), Cd5l (8.51), Fabp7 (8.42), C1qc (8.33), Prg4 (8.31), C3ar1 (8.27), Adamdec1 (8.07), Alox15 (8.07), Saa3 (7.86), Clec4n (7.76), Pf4 (7.66), C1qa (7.62), Fcgr4 (7.59), C6 (7.55), Gpnmb (7.44), Mmp13 (7.43), F5 (7.37), Slc40a1 (7.27), C1qb (7.25), Mertk (7.25), Cd300ld2 (7.13), Gbp2b (7.11), Ms4a4a (7.09), C5ar1 (7.03), Ms4a7 (6.91), Mafb (6.88), Ms4a14 (6.87), Klra2 (6.79), Alb (6.74), Fpr1 (6.73), Folr2 (6.65), Naip1 (6.6), Igha (6.53), Cbr2 (6.51), Ccl8 (6.5), Clec1b (6.48), Mrc1 (6.43), Cxcl13 (6.42), C4b (6.42), Ccl12 (6.41), Tfec (6.4), Adgre1 (6.38), Tlr8 (6.31), Clec4d (6.23), Olr1 (6.21), Pla2g2d (6.19), Hacd4 (6.1), Hal (6.1), Itgb5 (6.09), Cd163 (6.07), Slc11a1 (5.98), Lilra5 (5.95), Agmo (5.94), Chil3 (5.93), Pla2g7 (5.93), Slc7a2 (5.9), Slc7a8 (5.87), Fgd4 (5.85), Ccl24 (5.83), Fcgr1 (5.82), Abcd2 (5.81), Cd300ld (5.8), Ighv8-5 (5.79), Abcc3 (5.79), Msr1 (5.79), Ms4a6d (5.76), Igf1 (5.76), Fcna (5.76), Clec4a3 (5.75), Csf1r (5.71), Ccr1 (5.69), Ccr3 (5.68), Ms4a8a (5.68), Serpinb10 (5.66), Fpr2 (5.65), Hmox1 (5.63), Defa24 (5.63), Ptgs1 (5.62), Cd209g (5.59), Prss34 (5.58), Wfdc17 (5.58), Padi4 (5.55), Axl (5.54), Spic (5.53), Clec7a (5.5), Clec4f (5.46), Pilrb1 (5.45), Abca9 (5.43), Krt19 (5.43), Fabp1 (5.42), Ccl6 (5.41), Fcgr3 (5.4), Plau (5.4), Pilra (5.39), Cd209b (5.38), Reg3g (5.38), Ednrb (5.38), Siglec1 (5.36), Apoe (5.35), Plet1 (5.34), Serpina1a (5.31), Pmp22 (5.3), Lpl (5.28), F10 (5.26), Cd302 (5.26), Tbxas1

	(5.25), Spp1 (5.24), Car4 (5.22), Adgb (5.21), Lrp1 (5.2), Clec4a1 (5.2), Hpgds (5.18), Gm5150 (5.16), Hpgd (5.16), Etv1 (5.14), F13a1 (5.14), Mcpt8 (5.13), Ecm1 (5.11), Ifi204 (5.09), Dmxl2 (5.06), Gda (5.05), Igsf6 (5.04), Cd300ld4 (4.99), Pilrb2 (4.98), Npl (4.98), Cfh (4.97), Arg1 (4.97), Car1 (4.96), Cd14 (4.96), Siglece (4.94), Lyz2 (4.94), F7 (4.93), Ccl7 (4.91), Serpinb8 (4.9), Timd4 (4.89), Emilin2 (4.89), Il1a (4.89), Trf (4.89), Rnase4 (4.87), Ctsk (4.85), Clec4e (4.83), Mgst1 (4.82), Mmp9 (4.82), Apoc1 (4.82), Ighv1-84 (4.81), Gbgt1 (4.79), Alox5 (4.78), Slpi (4.78), Tlr13 (4.78), Serpina1c (4.76), Tgfb2 (4.75), Il10 (4.71), Ifi207 (4.69), Lipf (4.67), Clec5a (4.67), Gpx3 (4.66), Trem1 (4.65), Hgf (4.6), Cd33 (4.59), Nxpe5 (4.57), Lpcat2 (4.56), Cxcl2 (4.54), Il18 (4.53), Fn1 (4.53), Retnla (4.5), 1810011H11Rik (4.5), Tlr4 (4.48), Car6 (4.47), Lilr4b (4.46), Adgre4 (4.45), P2ry13 (4.43), Cmklr1 (4.43), Snx24 (4.42), Cd300lb (4.39), Ophn1 (4.39), Adap2 (4.38), Mfsd7c (4.36), Vstm2a (4.36), Frmd4b (4.33), Lrrc25 (4.3), Ighv3-5 (4.3), Aspa (4.28), Pcolce2 (4.26), Slc9a4 (4.25), Csf3r (4.25), Creg1 (4.25), Olfml3 (4.24), F630028O10Rik (4.24), Tmem106a (4.22), P2ry12 (4.22), Reg3b (4.22), Slc16a7 (4.22), Jchain (4.21), Rnase2a (4.19), Slc15a3 (4.19), Dpep2 (4.19), Ltc4s (4.19), Plxdc2 (4.18), Ccl2 (4.16), Pla2g15 (4.14), Plin2 (4.13), Pros1 (4.12), Oit3 (4.11), Ocstamp (4.11), Pla2g4a (4.11), Rin2 (4.1), Ifitm6 (4.1), Ltbp1 (4.1), Gc (4.09), Lilrb4a (4.09), Prg2 (4.09), Ear2 (4.09), Pdgfc (4.07), Fcrls (4.06), Camk1 (4.06), Mamdc2 (4.06), Nlrp3 (4.02), Cd300c2 (4.01), Ccl9 (4), Selp (4), Lgmn (4), Cyp2ab1 (3.99), Cfp (3.99), Dab2 (3.98), Ptgs2 (3.98), Kcnn3 (3.97), Sdc3 (3.97), Tlr12 (3.97), Serpinb2 (3.97), Paqr9 (3.97), Slfn4 (3.96), Tnfaip2 (3.95), Il1rn (3.94), Ptafr (3.94), Calml4 (3.93), Acp2 (3.93), Pparg (3.93), Wnt2 (3.93), Tc2n (3.92), Lyz1 (3.92), Cidec (3.91), Mmp19 (3.9), Edil3 (3.9), Il1b (3.9), Igkv8-21 (3.89), Dgki (3.89), Sash1 (3.89), Itsn1 (3.89), Hk3 (3.88), Pon3 (3.88), Cd200r4 (3.87), Fabp2 (3.87), Pltp (3.86), Kcnj10 (3.86), Mitf (3.85), Blvrb (3.85), Ninj1 (3.84), Lgals2 (3.84), Car3 (3.82), Siglecf (3.82), Spon1 (3.81), Cebpb (3.79), S100a1 (3.78), Tnfrsf21 (3.78), Mir99ahg (3.77), Acod1 (3.77), Reps2 (3.77), Mt1 (3.76), Rab31 (3.76), Sqrdl (3.75), Metrnl (3.75), P2rx4 (3.75), Nr1h3 (3.74), Cd300a (3.73), Ptgis (3.73), Renbp (3.72), Kcnj16 (3.72), Cln8 (3.72), Mmp27 (3.72), Rab3il1 (3.71), Vcan (3.69), Clec4a2 (3.69), Ctsg (3.68), Hcar2 (3.67), Pld1 (3.67), Cebpa (3.67), Tm4sf19 (3.66), Epb41l3 (3.66), Ly96 (3.66), Ctsb (3.66), Cd68 (3.66), Adgrl3 (3.64), Tlr5 (3.64), Mcemp1 (3.63), Gdpd3 (3.63), Ugt1a7c (3.63), Hfe (3.62), Muc3 (3.62), Aoah (3.61), BC018473 (3.6), Dhrs3 (3.6), Galc (3.6), B430306N03Rik (3.6), Fam46a (3.59), Chp2 (3.58), Stard8 (3.57), Bst1 (3.56), Hnmt (3.56), Sort1 (3.55), Pstpip2 (3.55), Loxl2 (3.54), Sh2d1b1 (3.54), Idh1 (3.54), Gas6 (3.5), Xdh (3.49), Fcgr2b (3.49), Mcpt4 (3.49), Itgad (3.48), Gatm (3.48), Aif1 (3.48), Lgals3 (3.48), Cysltr1 (3.48), Mcoln3 (3.47), Wdfy3 (3.47), Ccl3 (3.46), Fam20c (3.46), Itga9 (3.46), Fam20a (3.46), Lonrf3 (3.45), Msrb1 (3.45), Lacc1 (3.45), Ccrl2 (3.44), Plaur (3.44), Thbs1 (3.43), Dse (3.43), Gla (3.42), NA.81 (3.42), Plod3 (3.42), Nceh1 (3.42), Oas1g (3.41), Hexa (3.4), Pygl (3.4), Rhob (3.39), Rnf128 (3.39), Fam234a (3.39), Cryl1 (3.38), Lamp2 (3.38), Serpinb6a (3.38), Dnase2a (3.37),

	Selenop (3.37), Myof (3.37), P2ry6 (3.36), Slc38a6 (3.36), Tcn2 (3.35), Elane (3.35), Igkv4-57-1 (3.32), LOC100038947 (3.32), Apobec1 (3.31), Fgfr1 (3.31), Hebp1 (3.31), Stab1 (3.31), Tgfbi (3.3), Spint1 (3.3), Clmn (3.29), Cgnl1 (3.29), Maf (3.29), 6430548M08Rik (3.28), Aldh3b1 (3.28), Tcf7l2 (3.28), Itgam (3.28), Rasgef1b (3.27), Fabp5 (3.27), Vcam1 (3.26), Atp1a3 (3.26), 1700112E06Rik (3.26), Tlr2 (3.26), Cxcl9 (3.26), Arl11 (3.25), Tpd52 (3.25), Pcyox1 (3.25), Mmp12 (3.24), Tanc2 (3.24), Stard9 (3.24), Scamp5 (3.23), Etv5 (3.23), Cd59a (3.23), Colec12 (3.23), Cldn13 (3.22), Gm4951 (3.22), Tlr7 (3.21), Dnase1l1 (3.21), Ighv1-64 (3.21), Sirpa (3.21), Garnl3 (3.18), Perp (3.18), Igkv12-41 (3.17), Ctsc (3.16), Mgl2 (3.16), Slco2b1 (3.16), Asah1 (3.15), Mt2 (3.15), Man1c1 (3.15), Rab20 (3.14), Abhd12 (3.13), Vnn3 (3.12), Prtn3 (3.12), C130050O18Rik (3.12), Gstm1 (3.12), Smpdl3a (3.11), Slc6a4 (3.11), Htr2c (3.1), Mmp14 (3.09), Capns2 (3.09), Fcgrt (3.09), Timp2 (3.09), Naip6 (3.09), Ttyh2 (3.08), Zeb2 (3.08), Glul (3.08), Gdpd1 (3.08), Il1r2 (3.08), Ighv1-85 (3.08), Clec10a (3.06), Treml4 (3.06), Ifitm2 (3.06), Tmem26 (3.06), Igkv6-13 (3.05), Tmbim1 (3.03), Acer3 (3.03), Cybb (3.03), Rgl1 (3.03), Lipa (3.03), Ucp3 (3.02), Litaf (3.02), Mtmr7 (3.02), Lpar1 (3.02), Krt80 (3.01), Col14a1 (3.01), Naip5 (3.01)
NK cells	Klra8 (11.49), Klra7 (9.89), Ncr1 (9.59), Klra13-ps (8.15), NA.94 (8.08), Klri2 (8.04), Klrb1a (7.87), Klre1 (7.44), Klra6 (7.18), Klrb1c (7.02), Klrc3 (6.77), Klra4 (6.45), Adamts14 (6.3), Khdc1a (6.27), Itga2 (6.17), Cma1 (5.99), Styk1 (5.76), Klra1 (5.68), Samd3 (5.6), Klrc2 (5.56), Klra3 (5.55), Gzma (5.55), Prf1 (5.39), Eomes (5.13), Spry2 (5.12), Klra10 (5.05), Klrk1 (4.7), Serpinb9b (4.63), Klra5 (4.52), Fasl (4.51), Arl4d (4.43), Klf12 (4.41), Clnk (4.35), Klrb1f (4.29), Il12rb2 (4.21), Sytl3 (4.15), Gm6637 (4.11), Nrarp (4.08), Klrd1 (4.04), Il18rap (3.97), Trdv4 (3.95), Tbx21 (3.95), Gpx8 (3.77), Gzmb (3.76), Il2rb (3.74), Gm4956 (3.73), LOC102638466 (3.73), Cldnd2 (3.72), Pvrig (3.67), Osbpl3 (3.64), Klrg1 (3.61), S1pr5 (3.49), Car5b (3.46), Klrb1b (3.42), Klrc1 (3.36), Kcnip3 (3.3), Cd244 (3.3), Klrb1 (3.19), Cdc20b (3.17), Chsy1 (3.14), Dapk2 (3.11), Nkg7 (3.07), Arsb (3.06), Ctsw (3.06), Cym (3.05), Cyp17a1 (3.04)
T cells	Trac (8.6), Themis (8.35), Traj49 (7.54), Cd3g (7.52), Trav14d-3-dv8 (7.15), Dapl1 (7.09), Cd3e (6.95), Cd3d (6.68), Trav7-5 (6.56), Trav15-1-dv6-1 (6.18), Trav14-1 (6), Traj41 (5.99), Cd5 (5.96), Trat1 (5.88), Cd6 (5.86), Lat (5.67), Traj44 (5.34), Aqp11 (5.25), Trbv29 (5.15), Cd28 (5.11), Itk (4.91), Trav8n-2 (4.91), Tcrg-V4 (4.9), Camk4 (4.88), Tcf7 (4.84), Bcl11b (4.8), Nsg2 (4.78), Cd8b1 (4.65), Thy1 (4.59), Cxcr6 (4.57), Rorc (4.5), Il2ra (4.44), Ccr4 (4.43), Traj57 (4.36), Cd40lg (4.35), Gm14085 (4.25), Trav21-dv12 (4.24), Cd27 (4.21), Dzip1 (4.2), Lef1 (4.15), Trbv4 (4.15), Inpp4b (4.1), Ctla4 (4.08), Trav7-1 (4.06), Ccr8 (4.04), Izumo1r (3.85), Ubash3a (3.84), Cd96 (3.82), Ift80 (3.81), Arpp21 (3.79), Trbv1 (3.78), Slfn1 (3.78), Foxp3 (3.76), Dgkeos (3.74), Trgj4 (3.73), Sidt1 (3.71), Icos (3.68), Tnfsf8 (3.66), Ikzf2 (3.62), Il3 (3.6), Gzmk (3.59), Pdcd1 (3.58), Slc16a5 (3.57), 2010016I18Rik (3.51), Il2 (3.5), Tdrp (3.49), Itm2a (3.48), Lck (3.47),

	Tcrg-C4 (3.47), Skap1 (3.46), Fam78a (3.44), Dgka (3.32), Tox (3.31), Prkcq (3.3), Trav14n-3 (3.25), Trdv1 (3.23), Zap70 (3.2), Sit1 (3.19), Cd8a (3.16), Trav7d-4 (3.09), Cd247 (3.03), Tdrd5 (3.01), Mir181b-1 (3.01), LOC105246089 (3.01), Gata3 (3.01)

Supplementary Table 7. The gene sets used for cell typing the Visium spatial data of the B16F10 mouse model of melanoma.

References

1. Zheng,G.X.Y., Terry,J.M., Belgrader,P., Ryvkin,P., Bent,Z.W., Wilson,R., Ziraldo,S.B., Wheeler,T.D., McDermott,G.P., Zhu,J., *et al.* (2017) Massively parallel digital transcriptional profiling of single cells. *Nat. Commun.*, **8**, 14049.

2. Zhang,X., Lan,Y., Xu,J., Quan,F., Zhao,E., Deng,C., Luo,T., Xu,L., Liao,G., Yan,M., *et al.* (2019) CellMarker: a manually curated resource of cell markers in human and mouse. *Nucleic Acids Res.*, **47**, D721–D728.
